# Insertion sequence transposition activates antimycobacteriophage immunity through an *lsr2*‐silenced lipid metabolism gene island

**DOI:** 10.1002/mlf2.12106

**Published:** 2024-03-26

**Authors:** Yakun Li, Yuyun Wei, Xiao Guo, Xiaohui Li, Lining Lu, Lihua Hu, Zheng‐Guo He

**Affiliations:** ^1^ State Key Laboratory for Conservation and Utilization of Subtropical Agro‐Bioresources, Guangxi Research Center for Microbial and Enzyme Engineering Technology, College of Life Science and Technology Guangxi University Nanning China

**Keywords:** antiphage defense, bacteriophage, gene island, insertion sequence, mycobacteria

## Abstract

Insertion sequences (ISs) exist widely in bacterial genomes, but their roles in the evolution of bacterial antiphage defense remain to be clarified. Here, we report that, under the pressure of phage infection, the IS*1096* transposition of *Mycobacterium smegmatis* into the *lsr2* gene can occur at high frequencies, which endows the mutant mycobacterium with a broad‐spectrum antiphage ability. Lsr2 functions as a negative regulator and directly silences expression of a gene island composed of 11 lipid metabolism‐related genes. The complete or partial loss of the gene island leads to a significant decrease of bacteriophage adsorption to the mycobacterium, thus defending against phage infection. Strikingly, a phage that has evolved mutations in two tail‐filament genes can re‐escape from the *lsr2* inactivation‐triggered host defense. This study uncovered a new signaling pathway for activating antimycobacteriophage immunity by IS transposition and provided insight into the natural evolution of bacterial antiphage defense.

## Impact statement

Very few antiphage mechanisms have been clearly characterized in mycobacterial species, although these bacterial genomes encode potential adaptive and innate immune systems. In this study, we show that endogenous insertion sequences in mycobacteria can activate the defensive gene islands, thereby helping bacteria quickly acquire a broad‐spectrum antiphage ability in a short period of time. This may represent a new acquired defense strategy different from the previously reported CRISPR antiphage mechanism.

## INTRODUCTION

Insertion sequences (ISs), the highly prevalent mobile genetic elements in bacterial genomes, play important roles in triggering genomic evolution and bacterial adaption to adverse environments including bacteriophage infection^1^, ^2^, ^3^. Bacteriophages or phages are a group of viruses that specifically infect bacteria, and widely exist in natural and human environments^4^, ^5^, ^6^. Unavoidably, host bacteria are under attack by their phages, and thus need to continuously develop new antiphage strategies for survival. However, there are only sporadic reports on the role of ISs in the evolution of bacterial antiphage defense^3^, ^7^, and the involved regulatory mechanisms of ISs remain largely unclear.

Phages are obligate parasites that exploit host bacterial functions for propagation. The process of their infection mainly involves adsorption, injection, replication, lysis, and release^8^. Phage adsorption relies on highly specific interactions between phage receptor‐binding protein and bacterial receptors. Bacterial receptors can be any components exposed to the cell surface, such as membrane surface‐anchored protein^9^, extracellular polysaccharides^10^, ^11^, and flagella^12^. Faced with phage infection, host bacteria can produce extracellular matrix to hinder phage adsorption and prevent receptor recognition through receptor mutations or deletions to produce phage resistance^13^. In addition, bacteria can cleave or degrade DNA by recognizing specific sequence motifs on the phage genomes through defense systems such as Dnd^14^, DISARM^15^, and CRISPR‐Cas^16^. Very recently, it has been shown that some antiphage defense systems of bacteria consume molecules necessary for virus replication to resist the invasion of phages^17^, ^18^, ^19^.

In the arm race between phages and their hosts, phages have evolved a series of antidefense strategies. For example, many phages encode anti‐CRISPRs (Acrs) proteins that can counteract the bacterial CRISPR‐Cas immune response^20^. Some phages encode anti‐CBASS (Acb) and anti‐Pycsar (Apyc) proteins, specifically degrading cyclic nucleotide signals that activate host immunity to counteract defense^21^ or encoding antichelating cyclic nucleotide signaling proteins to overcome phage defense^22^.

Mycobacteria represent a large group of important actinomycetes, among which *Mycobacterium tuberculosis*, which causes human tuberculosis, is the most well known. In addition, it includes the nonpathogenic and rapidly growing *Mycobacterium smegmatis*, which is often used as a model strain for studying genetic mechanisms of mycobacterial species^23^. Mycobacterial genomes contain abundant IS elements, of which IS*6110* has been widely used as a marker in the molecular epidemiology of tuberculosis^24^, and its transposition can help produce antibiotic resistance^25^; IS*1096* is considered unique to *M. smegmatis* ^26^, and plays a crucial role in mycobacterial genome evolution^27^. Recently, it has been found that mycobacterial genomes encode some potential immune systems that resist phage infection, including the CRISPR‐Cas system^28^, the toxin–antitoxin system^29^, the restriction–modification system^29^, and the BREX‐like system^30^. However, their roles in phage defense are still largely unclear. Especially, there are very few reports on the evolution of mycobacterial defense against phages and related regulatory pathways through IS transposition remain to be explored.

Lsr2 is a conserved histone‐like nucleoid‐associated protein and widely exists in mycobacteria and other actinobacteria^31^. Its N‐terminal domain participates in oligomerization, allowing Lsr2 to form nucleoprotein complexes^32^, ^33^, and the C‐terminal domain is responsible for targeting AT‐rich sequences in genomic DNA^31^. In *M. smegmatis* and *M. tuberculosis*, Lsr2 directly or indirectly controls the transcription of many genes and participates in multiple cellular processes including cell wall biosynthesis^34^, ^35^, ^36^. For example, Kołodziej et al. utilized ChIP‐seq and quantitative real‐time (qRT)‐PCR assays to confirm that Lsr2 directly binds to the *MSMEG_4727* upstream region of an Lsr2‐silenced lipooligosaccharides (LOS) synthesis gene island, and negatively regulates the expression of *MSMEG_4727*, which encodes a mycocerosic acid synthase‐like polyketide synthase in *M. smegmatis* ^37^. Very recently, the function of *lsr2* gene has also been connected to the phage DNA replication^38^.

In this study, we found that, upon mycobacteriophage infection into *M. smegmatis*, IS*1096* can translocate and insert into the *lsr2* gene at high frequencies, activating the expression of the LOS metabolism gene cluster composed of 11 genes, *MSMEG_4727–4737* designated as the LOS synthesis gene island^39^, which endows the host with a broad resistance to mycobacterial phages. Furthermore, we have successfully isolated a phage that has evolved mutations in its tail‐filament genes, and can re‐escape from the mycobacterial defense. This study uncovered a previously undefined signaling pathway for IS transposition to activate antimycobacteriophage immunity and provided evidence to show that an IS can trigger the co‐evolution of host mycobacterial antiphage defense and its phage's antidefense.

## RESULTS

### IS*1096* transposition into the *lsr2* gene of *M. smegmatis* produces a broad antiphage activity

**Figure 1:**
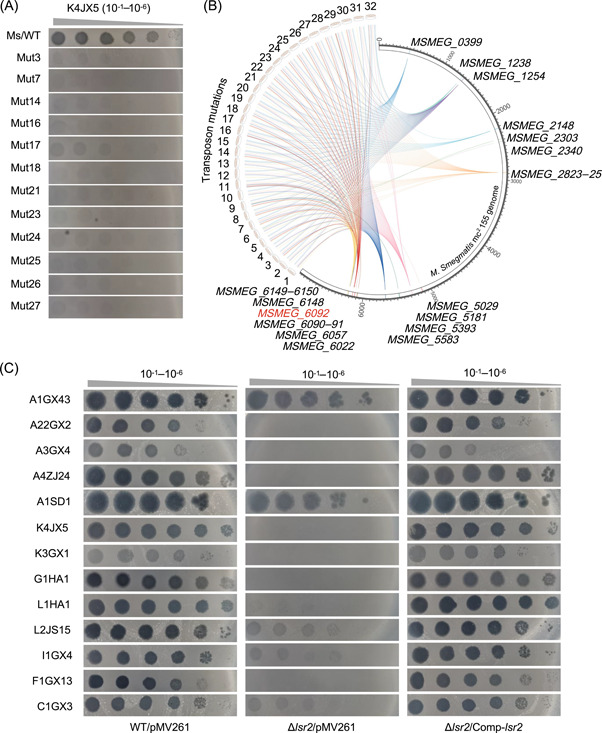
IS*1096* transposition into the *lsr2* gene of *Mycobacterium smegmatis* produces a broad antiphage phenotype. (A) Resistant assays for 12 representative mutant *M. smegmatis* strains to the infection of phage K4JX5. The K4JX5 dilution shown at the top was spotted on mycobacterial cell lawns. Names of 12 mutant strains formed by different types of insertion sequences (ISs) in the genome of *M. smegmatis* (Mut3‐27) are shown on the left. The antiphage phenotypes of the other 20 mutant strains are shown in Figure S1A. (B) Circular genetic map of IS types of 32 mutant strains and their insertion sites in the genome DNA of *M. smegmatis*. A series of endogenous ISs and their corresponding inserted genes in the genome of these mutant strains are indicated. In 20 of all 32 mutant strains, the IS*1096* was found to be reinserted into the *lsr2* gene (*MSMEG_6092*) indicated by red print. (C) Plaque formation ability assays for multiple mycobacteriophages on the lawns of three different *M. smegmatis* strains. WT/pMV261, Δ*lsr2*/pMV261, or Δ*lsr2*/Comp‐*lsr2* represent the wild‐type, *lsr2* deletion, and *lsr2‐*complemented strains, respectively. Phage names are shown on the left.

To characterize antiphage growing mutant strains, we first performed a screen on a previously constructed TM4‐inserted mutant *M. smegmatis* library^40^ under the pressure of mycobacteriophage infection. As shown in Figures 1A and S1A, 32 mycobacterial strains resistant to K4JX5 infection were successfully isolated and complete genomes of all these strains were subsequently sequenced. A further comparative genomic analysis found that there is a TM4 insertion mutant position in the genome of each of these strains (Table S1). Unexpectedly, a series of endogenous IS transpositions also occurred in these mutant strains that disrupt 14 genes including the *lsr2* gene (Table S2). In particular, the IS*1096* was found to be reinserted into the *lsr2* gene in 20 of all 32 strains (Figure 1B and Table S1). We thereafter utilized a CRISPRi strategy to show that only the mutation of the *lsr2* gene translocated by IS*1096*, but not the 13 mutated genes by endogenous IS transpositions (Figure S1B) or any gene inactivated by TM4 insertion (Figure S1C) in these 20 strains, is most likely responsible for the phage‐resistant phenotype.

Next, we confirmed that the *lsr2* deletion resulted in a broad‐spectrum antiphage phenotype of *M. smegmatis*. The *lsr2* gene was knocked out by using a recombination strategy and its complementary strain was also constructed by transforming a recombinant plasmid containing the wild‐type *lsr2* gene into the deletion strain. As shown in Figure 1C, compared with the wild‐type *M. smegmatis* strain, which is sensitive to various clusters of mycobacteriophages (left panel), the *lsr2* deletion strain acquired clear resistance to the infection of multiple phages including clusters A3GX4, A4ZJ24, A22GX2, K3GX1, K4JX5, G1HA1, L1HA1, I1GX4, and F1GX13 (middle panel). Strikingly, all these phages re‐acquired original infection ability to the complementary strain under a similar condition (right panel), indicating that the *lsr2* mutation is responsible for the antiphage phenotype of these isolated resistant strains.

Taken together, our results showed that the IS*1096* transposition into the *lsr2* gene occurs at high frequencies under the pressure of phage infection, and the *lsr2*‐mutated mycobacterium acquires broad‐spectrum antiphage ability.

### The LOS synthesis gene island significantly contributes to the antiphage ability of the *lsr2* deletion strain

**Figure 2:**
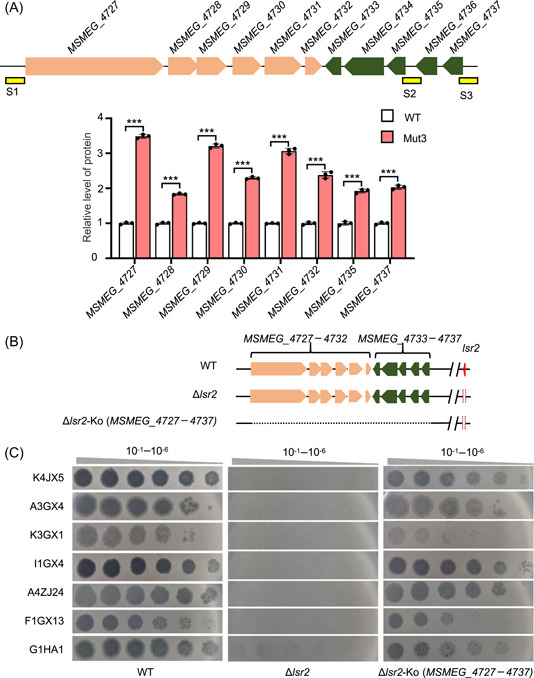
The lipooligosaccharides (LOS) synthesis gene island confers the phage resistance of the *lsr2* deletion strain. (A) Assays for effects of the *lsr2* insertion mutant on the expression of the LOS synthesis genes in *Mycobacterium smegmatis*. Upper, schematic of the LOS synthesis gene island. Lower, proteomic assays for the protein expression difference of the LOS synthesis gene island between wild‐type (WT) and Mut3 strains. WT and Mut3 represent the WT strain and *lsr2*‐inactivated strain, respectively. Data indicate mean ± SD from three replicates and were analyzed using an unpaired two‐tailed Student's *t* test (***p < 0.001). (B) Schematic diagrams depicting deletion of the LOS synthesis gene island. The dashed line indicates the corresponding gene deletion. Δ*lsr2*‐Ko (*MSMEG_4727–4737*) represents a co‐deleted strain of both *lsr2* and the *MSMEG_4727–4737* gene island. (C) Effects of co‐deleting the LOS synthesis gene island on the sensitivity of the *lsr2* deletion strain to mycobacteriophages. The co‐deleted strain re‐acquired a sensitive phenotype to the infection of multiple different clusters of mycobacteriophages.

To pursue the *lsr2* inactivation‐triggered pathway, a comparative proteomic analysis was utilized to search differential genes potentially responsible for the antiphage ability of the *lsr2* deletion strain. When compared with expression in the wild‐type *M. smegmatis* strain, 39 proteins (log_2_FC ± 0.8 and *p* < 0.05) in the *lsr2‐*mutated strain translocated by IS*1096*, Mut3, were found to have significant expression differences (Figure S2); these include 30 upregulated proteins and 9 downregulated proteins (Figure S2B). Interestingly, an overall upregulation was observed for a previously defined LOS synthesis‐related gene cluster composed of 11 genes (Figure 2A and Figure S2B, upper panel) (Table S3), designated here as the LOS synthesis gene island, which includes genes from *MSMEG_4727* to *MSMEG_4737* (Figure 2A, upper panel).

Next, we performed knockout analysis of the complete LOS synthesis gene island to examine whether it contributes to antiphage defense in the mycobacterium. Using a recombination strategy, the LOS synthesis gene island, *MSMEG_4727–4737*, was successfully co‐deleted in the *lsr2* deletion *M. smegmatis* strain (Figure 2B). As shown in the right panel of Figure 2C, the co‐deleted strain re‐acquired a sensitive phenotype to the infection of multiple different clusters of mycobacteriophages, which is very close to the phenotype of the wild‐type strain (Figure 2C, left panel) and in contrast to the phenotype of the *lsr2* deletion strain (Figure 2C, middle panel).

These results suggest that the LOS synthesis gene island significantly contributes to the *lsr2* deletion‐triggered resistance to infection of multiple clusters of mycobacterial phages.

### Lsr2 directly regulates expression of the LOS synthesis gene island

**Figure 3:**
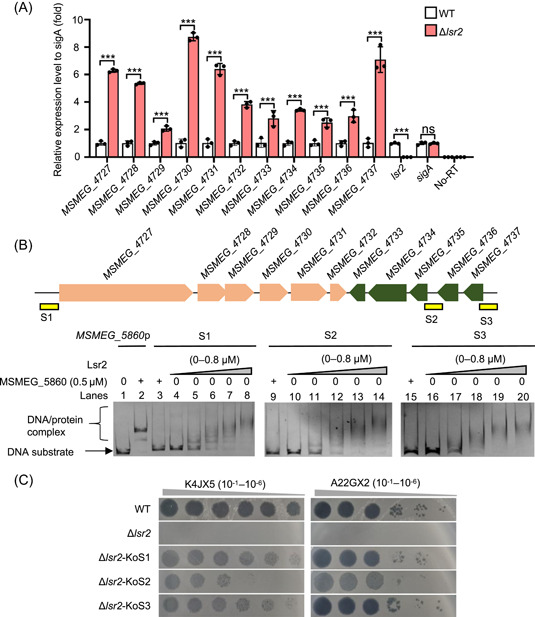
Lsr2 directly regulates expression of the LOS synthesis gene island. (A) Quantitative real‐time‐PCR (qRT‐PCR) assays for the expression difference of the LOS synthesis island genes between the WT and Δ*lsr2* strains of *Mycobacterium smegmatis*. *sigA* was used as a control. (B) EMSA assays for the binding ability of Lsr2 to the three upstream DNA substrates. The noncoding regions, recognized by Lsr2, are indicated by a yellow box. S1 (lanes 5–8), S2 (lanes 11–14), and S3 (lanes 17–20) were co‐incubated with various amounts of Lsr2; lanes 4, 10, and 16 represent three DNA substrate blank controls without protein. MSMEG_5860 was used as a negative control protein (lanes 3, 9, and 15) and incubated with *MSMEG_5860*p (lane 2). The free DNA substrate and DNA–protein complex are indicated in the left panel. (C) Assays for the plaque formation ability of two mycobacteriophages, K4JX5 and A22GX2, by spotting onto the lawns of co‐deleted *M. smegmatis* strains of *lsr2* and different DNA substrate. Δ*lsr2*‐KoS1, Δ*lsr2*‐KoS2, and Δ*lsr2*‐KoS3 represent the S1/2/3 and *lsr2* co‐deleted strains, respectively, and are indicated in the left panel. Data were analyzed using an unpaired two‐tailed Student's *t* test (***p < 0.001).

The *lsr2* encodes a histone‐like nucleoid‐associated protein that considerably affects gene transcription in mycobacteria, and a previous study has confirmed that Lsr2 regulates expression of certain genes of the island^35^, ^36^, ^37^. Consistently, using a qRT‐PCR assay as shown in Figure 3A, we confirmed that all genes within the LOS synthesis gene island were significantly upregulated in the *lsr2* deletion strain (*p* < 0.01), and were downregulated in the *lsr2*‐overexpressing strain (*p* < 0.01) (Figure S3) compared to those in the wild‐type strain.

Next, an electrophoretic mobility shift assays (EMSA) was utilized to examine if the purified Lsr2 protein can directly bind to the upstream DNA fragment of the island genes. As shown in Figure 3B, a stepwise increase in the amount of shifted DNA substrate S1 was observed with increasing amounts of the Lsr2 protein (0–0.8 μM) in the reactions, and a gradual appearance of slow‐mobility complexes was observed (lanes 5–8). In contrast, no DNA‐binding activity could be observed for the control protein MSMEG_5860 under the same experimental conditions (Figure 3B, lane 3). A similar DNA‐binding ability of Lsr2 can also be observed for two other upstream DNA fragments: S2 and S3 (Figure 3B). Therefore, our results showed that Lsr2 could directly bind with the upstream regulatory sequences of the LOS synthesis gene island.

We further evaluated the effects of these three upstream regulatory sequences on the mycobacterial antiphage activity in vivo in the *lsr2* deletion strain. Using a similar recombination strategy to that described above, the three upstream DNA fragments, S1, S2, and S3 (Figure 3B, upper panel), were successfully knocked out, respectively, in the *lsr2* deletion strain. As shown in Figure 3C, all three co‐deleted strains, Δ*lsr2*/KoS1, Δ*lsr2*/KoS2, and Δ*lsr2*/KoS3, lost their antiphage phenotypes and re‐acquired sensitivity to the infection of either phage K4JX5 or A22GX2, which is very similar to the phenotype of the wild‐type strain.

Taken together, these results showed that the three upstream regulatory sequences of the LOS synthesis gene island are required for the *lsr2* inactivation‐triggered antiphage activity, and Lsr2 can directly bind to these regulatory sequences and negatively regulates expression of the LOS synthesis island genes.

### Most of the LOS synthesis island genes are required for retaining the antiphage phenotype of the *lsr2* deletion strain

**Figure 4:**
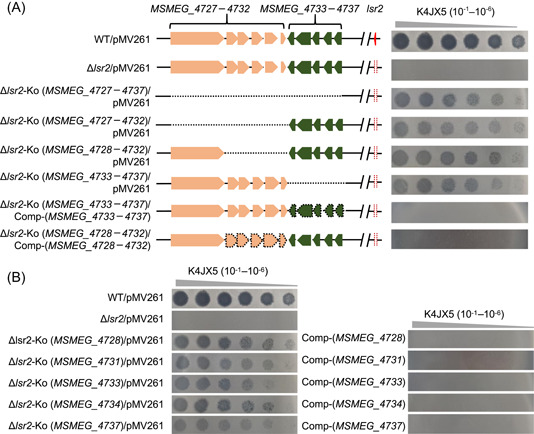
Most of the LOS synthesis island genes are required for retaining the antiphage phenotype of the *lsr2* deletion strain. (A) Effects of complete or partial knockout of the LOS synthesis gene island on the resistance of the *lsr2* deletion strain to phage JXK4_5. Left, the diagram of deletion of the LOS synthesis gene island or its partial genes. Right, the resistance of different recombinant *Mycobacterium smegmatis* strains to phage K4JX5. Δ*lsr2*‐Ko (*MSMEG_4727–4737*)/pMV261, Δ*lsr2*‐Ko (*MSMEG_4727–4732*)/pMV261, Δ*lsr2*‐Ko (*MSMEG_4728–4732*)/pMV261, and Δ*lsr2*‐Ko (*MSMEG_4733–4737*)/pMV261, represent the co‐deleted strains of *lsr2* and different regions of the LOS synthesis gene island. Δ*lsr2*‐Ko (*MSMEG_4733–4737*)/Comp‐(*MSMEG_4733–4737*) and Δ*lsr2*‐Ko (*MSMEG_4728–4732*)/Comp‐(*MSMEG_4728–4732*) represent the corresponding complementary strains of complete or partial deletion of the LOS synthesis gene island, respectively. (B) Effects of co‐deleted a single gene of the LOS synthesis gene island in the Δ*lsr2* strain on the plaque formation of phage K4JX5 by spotting phage on the lawns of different recombinant *M. smegmatis* strains. Δ*lsr2*‐Ko (*MSMEG_4728*)/pMV261, Δ*lsr2*‐Ko (*MSMEG_4731*)/pMV261, Δ*lsr2*‐Ko (*MSMEG_4733*)/pMV261, Δ*lsr2*‐Ko (*MSMEG_4734*)/pMV261, and Δ*lsr2*‐Ko (*MSMEG_4737*)/pMV261 represent the co‐deleted strains of *lsr2* and a corresponding single gene of the LOS synthesis gene island, respectively. Comp‐(*MSMEG_4728*), Comp‐(*MSMEG_4731*), Comp‐(*MSMEG_4733*), Comp‐(*MSMEG_4734*), and Comp‐(*MSMEG_4737*) represent the corresponding complementary strain of their corresponding gene of the LOS synthesis gene island, respectively.

Next, the mycobacteriophage K4JX5 was utilized as an example to identify the potential antiphage pathway in the *lsr2* deletion strain. We first constructed three co‐deleted strains, including Δ*lsr2*‐Ko (*MSMEG_4727–4732*), Δ*lsr2*‐Ko (*MSMEG_4728–4732*), and Δ*lsr2*‐Ko (*MSMEG_4733–4737*), in which partial genes of the LOS synthesis island were deleted (Figure 4A, left panel). Strikingly, all three co‐deleted strains re‐acquired a sensitive phenotype to the mycobacteriophage K4JX5 infection, which is very close to that of the complete LOS synthesis gene island‐deleted strain, Δ*lsr2*‐Ko (*MSMEG_4727–4737*) (Figure 4A, right panel). In contrast to their knockout strains, two complementary strains, Δ*lsr2*‐Ko (*MSMEG_4728–4732*)/Comp‐(*MSMEG_4728–4732*) and Δ*lsr2*‐Ko (*MSMEG_4733–4737*)/Comp‐(*MSMEG_4733–4737*), were resistant to the infection of K4JX5. A similar result could also be observed when another phage A22GX2 was spotted on the plate containing these different recombinant strains (Figure S4A). These results suggest that each part of the LOS synthesis gene island is essential for retaining the antiphage phenotype of the *lsr2* deletion strain.

Next, we constructed a series of co‐deleted strains, in which each of five LOS synthesis island genes was deleted, respectively, including Δ*lsr2*‐Ko (*MSMEG_4728*), Δ*lsr2*‐Ko (*MSMEG_4731*), Δ*lsr2*‐Ko (*MSMEG_4733*), Δ*lsr2*‐Ko (*MSMEG_4734*), and Δ*lsr2*‐Ko (*MSMEG_4737*). Five corresponding complementary strains were also successfully constructed. Clearly, deletion of each single gene could endow the *lsr2* deletion strain with a sensitive phenotype to the infection of mycobacteriophage K4JX5 (Figure 4B, left panel) or A22GX2 (Figure S4B, left panel). In contrast, all five complementary strains re‐acquired clear resistance to the infection of K4JX5 or A22GX2, which is very similar to the phenotype of the *lsr2* deletion strain (Figures 4B and S4B, right panel).

Taken together, these results suggest that most of the LOS synthesis island genes are required for the *lsr2* inactivation‐triggered pathway to fight against phages K4JX5 and A22GX2.

### The LOS synthesis gene island triggers abnormal phosphatidylinositol mannoside (PIM) accumulation in the *lsr2* deletion strain

**Figure 5:**
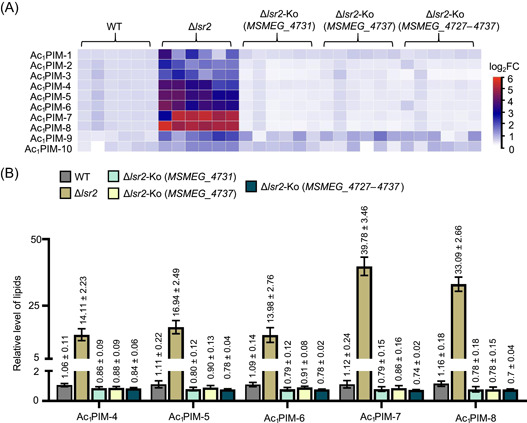
The LOS synthesis gene island triggers abnormal phosphatidylinositol mannoside (PIM) accumulation in the *lsr2* deletion strain. (A) Heatmaps showing differential profiles of Ac_1_PIM lipids in WT, Δ*lsr2*, Δ*lsr2*‐Ko (*MSMEG_4731*), Δ*lsr2*‐Ko (*MSMEG_4737*), and Δ*lsr2*‐Ko (*MSMEG_4727–4737*) strains determined by lipidomic assays. Ac_1_PIM‐1–10 represents Ac_1_PIM_4_ (R1CO2H+R2CO2H+R3CO2H=46:1, R4=H), Ac_1_PIM_4_ (R1CO2H+R2CO2H+R3CO2H=49:2, R4=H), Ac_1_PIM_4_ (R1CO2H+R2CO2H+R3CO2H=49:1, R4=H), Ac_1_PIM_4_ (R1CO2H+R2CO2H+R3CO2H=49:4, R4=H), Ac_1_PIM_4_ (R1CO2H+R2CO2H+R3CO2H=48:3, R4=H), Ac_1_PIM_3_ (R1CO2H+R2CO2H+R3CO2H=53:5, R4=H), Ac_1_PIM_4_ (R1CO2H+R2CO2H+R3CO2H=48:1, R4=H), Ac_1_PIM_3_ (R1CO2H+R2CO2H+R3CO2H=53:4, R4=H), Ac_1_PIM_2_ (R1CO2H+R2CO2H+R3CO2H=53:3, R4=H), and Ac_1_PIM_2_ (R1CO2H+R2CO2H+R3CO2H=51:1, R4=H), respectively. (B) Relative levels of 5 Ac_1_PIM lipids in Δ*lsr2*, Δ*lsr2*‐Ko (*MSMEG_4731*), Δ*lsr2*‐Ko (*MSMEG_4737*), and Δ*lsr2*‐Ko (*MSMEG_4727–4737*) compared with WT. Data represent mean ± SD (*n* ≥ 5).

Previous studies have suggested that both the *lsr2* gene and the LOS synthesis gene island are involved in the lipid metabolism in *M. smegmatis*, and the synthesis and localization of glycolipids or phospholipids affect the adsorption capacity of mycobacteriophages^37^, ^41^, ^42^, ^43^, ^44^, ^45^. To further address this issue, we first performed lipidomic analysis and compared the relative lipid composition of several recombinant strains, including wild‐type, Δ*lsr2*, Δ*lsr2*‐Ko (*MSMEG_4731*), Δ*lsr2*‐Ko (*MSMEG_4737*), and Δ*lsr2*‐Ko (*MSMEG_4727–4737*) strains. As shown in Figure S5A, changes in the contents of 300 lipid molecules in the Δ*lsr2* strain (multiple ≥2 and *p* < 0.05) were clearly observed. Strikingly, the levels of 10 different phosphatidylinositol mannosides (PIMs), which were designated from Ac_1_PIM‐1 to Ac_1_PIM‐10, respectively, were significantly upregulated in the Δ*lsr2* strain as shown in Figures 5 and S5B compared to those in the wild‐type strain. Among these several lipids, the content of Ac_1_PIM‐7 in the Δ*lsr2* strain changed most significantly (39.78 ± 3.46), which was 40‐fold higher than that in the wild‐type strain (1.12 ± 0.24). The change in Ac_1_PIM‐8 (33.09 ± 2.66) was also 33‐fold higher in the Δ*lsr2* strain than that in the wild type. When the complete LOS synthesis gene island or a single gene, either *MSMEG_4731* or *MSMEG_4737*, was co‐deleted in the Δ*lsr2* strain, the contents of all these Ac_1_PIMs in the recombinant strains were found to return to a level similar to that in the wild‐type strain (Figure 5B), indicating that the LOS synthesis gene island is required for abnormal PIM accumulation triggered by *lsr2* deletion.

Therefore, our results, together with those of previous reports, suggest that the *lsr2* gene plays a critically important role in maintaining mycobacterial lipid hemostasis, and the LOS synthesis gene island triggers abnormal PIM accumulation in the cell envelope of the *lsr2* deletion strain.

### The LOS synthesis gene island endows the *lsr2* deletion strain with a defect in K4JX5 phage absorption

**Figure 6:**
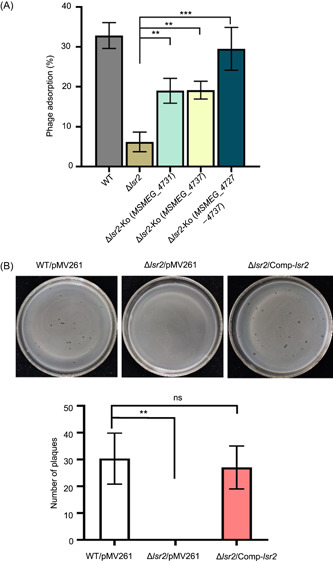
The LOS synthesis gene island endows the *lsr2* deletion strain with a defect in K4JX5 phage absorption. (A) Comparative assays for the adsorption of phage K4JX5 to different mycobacterial strains, including WT, Δ*lsr2*, Δ*lsr2*‐Ko (*MSMEG_4731*), Δlsr2‐Ko (*MSMEG_4737*), and Δ*lsr2*‐Ko (*MSMEG_4727–4737*). Bar graphs show phage adsorption after 40 min. (B) DNA electroporation assays for determining if the phage genomic DNA can complete the subsequent life cycle after entering the mycobacterial cell. The extracted genome of phage K4JX5 was transferred into the *lsr2* deletion strain by electroporation. After 1 h incubation, this sample was mixed with the WT/pMV261, Δ*lsr2*/pMV261, and Δ*lsr2*/Comp‐*lsr2* strains, and the number of phage plaques was then assessed. Data indicate mean ± SD from three replicates. ns, no significant difference; **p < 0.01; ***p < 0.001; unpaired two‐tailed Student's *t* test.

To further investigate the contribution of this gene island to *lsr2* inactivation‐triggered antiphage activity, we determined the phage absorption capacity of several recombinant strains, including Δ*lsr2*, Δ*lsr2*‐Ko (*MSMEG_4731*), Δ*lsr2*‐Ko (*MSMEG_4737*), and Δ*lsr2*‐Ko (*MSMEG_4727–4737*). As shown in Figure 6, we found that the *lsr2* deletion resulted in a nearly 6.5‐fold reduction in K4JX5 phage adsorption on the surface of the recombinant strains compared with that of the wild‐type strain. Strikingly, a nearly fourfold increase in phage adsorption on the co‐deleted strain of either *MSMEG_4731* (19.2%) or *MSMEG_4737* (19.5%) within the LOS synthesis gene island was observed compared with that on the *lsr2* deletion strain (4.7%). More interestingly, co‐deletion of the LOS synthesis gene island Δ*lsr2*‐Ko (*MSMEG_4727–4737*) (29%) resulted in a nearly sixfold increase in phage adsorption compared with that of the *lsr2* deletion strain, which is very close to the capacity of the wild‐type strain (32.5%). Therefore, we confirmed that *lsr2* deletion significantly inhibits K4JX5 phage adsorption on the mycobacterial cell surface and the LOS synthesis gene island is required for retaining the absorption defect phenotype of the *lsr2* deletion strain.

We next determined the amount of phage genomic DNA in both wild‐type and *lsr2* deletion strains during infection. At 80 min postinfection, the abundance value of phage DNA of the *lsr2* deletion strain was only 5.1 (Figure S6), whereas that in the wild‐type strain was nearly 5.2‐fold higher (26.04) than that in the mutant strain, which is consistent with the adsorption inhibition described above (Figure 6A).

To further investigate the *lsr2* inactivation‐triggered antiphage mechanism, we performed electroporation experiments to examine if the phage genomic DNA can complete the subsequent life cycle after entering the mycobacterial cell. As shown in Figure 6B, when the genomic DNA of phage K4JX5 was electroporated into the Δ*lsr2* strain and subsequently cocultured with, respectively, three different strains including the wild‐type strain, the Δ*lsr2* strain, and the Δ*lsr2*/Comp‐*lsr2* strain, about 30 phage plaques were observed on each double‐layer plate of the wild‐type strain and about 27 plaques were observed on the plate of the Δ*lsr2*/Comp‐*lsr2* strain (Figure 6B, lower panel). In contrast, no plaque can be observed on the plate of Δ*lsr2* strain because of a phage absorption defect in the subsequent infecting cycle, indicating that, once phage genomic DNA enters into the mycobacterial cells, it can complete its subsequent replication cycle and produce normal phage particles.

Therefore, our results showed that *lsr2* deletion resulted in a phage K4JX5 absorption defect and the LOS synthesis gene island was required for maintaining the defect phenotype of the *lsr2* deletion strain.

### A mutant phage with restored adsorption capacity re‐escapes the *lsr2* inactivation‐triggered defense

**Figure 7:**
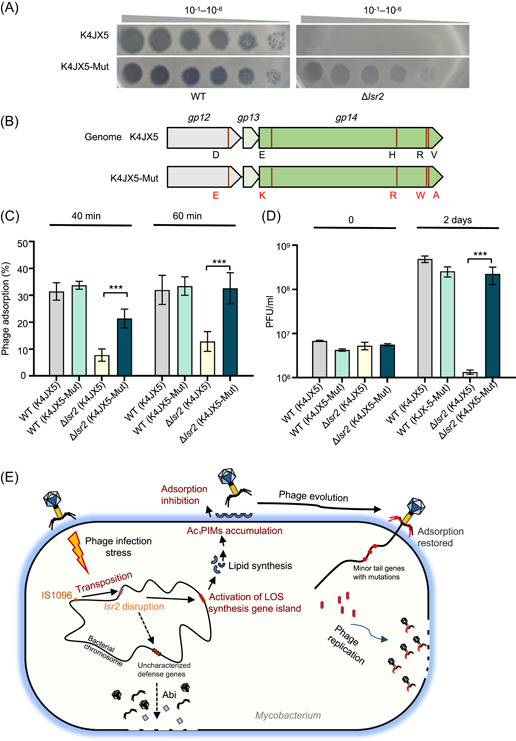
Mutant of phage K4JX5 escapes the antiphage defense of the *lsr2* deletion strain. (A) Comparative assays for the plaque formation ability of K4JX5 and its mutant (K4JX5‐Mut). Ten‐fold serial dilutions of phage K4JX5 and K4JX5‐Mut were spotted onto the lawns of the WT and Δ*lsr2* strains. (B) Locations of amino acid substitutions in the genome of K4JX5‐Mut are indicated by red lines. (C) Analysis of the adsorption difference between K4JX5 and K4JX5‐Mut phages to the WT and *lsr2* deletion strains at the time points of 40 min and 60 min. (D) Assays for the number of progeny phages produced by phages K4JX5 and K4JX5‐Mut during infection of WT and Δ*lsr2* strains, respectively. PFU/ml indicates the plaque‐forming unit. Data represent mean ± SD from three replicates.  ***p < 0.001; unpaired two‐tailed Student's *t* test. (E) Summary of the antiphage defense mechanisms triggered by *lsr2* deletion in *Mycobacterium smegmatis*. Upon phage infection, the IS*1096* is induced to reinsert into the *lsr2* gene, and the Lsr2‐silenced LOS synthesis gene island is subsequently activated. This triggers abnormal PIM accumulation in the cell envelope of the *lsr2* deletion strain, which results in the phage absorption defect of the mycobacterium, defending against phage infection. An evolved phage with mutations in its tail filament proteins, which restores its adsorption ability, can re‐escape the defense of the *lsr2* deletion strain.

To further confirm the importance of a phage absorption defect in the *lsr2* inactivation‐triggered antiphage pathway, we first tried to create a mutant phage that can escape from the defense of the *lsr2* deletion strain. As shown in Figure 7A, a mutant phage, K4JX5‐Mut, was successfully isolated by repeatedly spotting K4JX5 on the double‐layer plate containing the *lsr2* deletion strain. As the concentration of phages increased, the invading spots formed by K4JX5‐Mut became clearer (Figure 7A, lower right panel). In contrast, no transparent spot on the plate was observed for K4JX5 under the same conditions (Figure 7A, upper right panel). A further sequencing assay showed that K4JX5‐Mut had evolved several mutations in its two tail‐filament genes, *gp12* and *gp14*, which led to changes of five amino acids residues as shown in Figure 7B.

Next, we determined the differential absorption capacities of these two phages, K4JX5 and K4JX5‐Mut, on the wild‐type and *lsr2* deletion strains, respectively. As shown in Figure 7C, left panel, clear phage absorption to the wild‐type mycobacterial strain was observed for both phages at two different cocultured time points, 40 and 60 min. However, when these two phages were cocultured with the *lsr2* deletion strain under a similar condition, only a very low absorption rate (<10%) can be observed for K4JX5. Strikingly, a nearly 20% adsorption rate at 40 min was observed for K4JX5‐Mut, and the rate at 60 min (28%) was very close to that on the wild‐type mycobacterial strain. These results showed that the mutant phage K4JX5‐Mut re‐acquired good absorption capacity to the *lsr2* deletion strain, which is consistent with the fact that the phage has evolved mutations in the tail‐filament genes. We further determined the infection ability of K4JX5‐Mut for the *lsr2* deletion strain. As shown in Figure 7C, right panel, K4JX5‐Mut could effectively produce plaques on the plate of the *lsr2* deletion strain (10^8^), indicating that it acquired very good infection ability, which is very similar to the activity that the phage confers to the wild‐type strain. However, when infected with similar phage titers, very few plaques were observed for K4JX5 on the plate of the *lsr2* deletion strain, which is consistent with our result shown above (Figures 6A and 7A).

Taken together, our results showed that a mutant phage K4JX5‐Mut with good absorption capacity had successfully evolved mutations in tail‐filament genes and could escape from *lsr2* inactivation‐triggered host defense.

## DISCUSSION

Very few antiphage mechanisms have been clearly characterized in mycobacterial species, although these bacterial genomes encode potential adaptive immune systems such as the CRISPR‐Cas system^28^ and some innate immune systems^29^, ^30^. In this study, we found that, under the pressure of phage infection, the transposition of IS*1096* into the *lsr2* gene endowed *M. smegmatis* with a broad‐spectrum antiphage ability. Our data support the model shown in Figure 7E, in which, following phage infection, IS transposition in *M. smegmatis* is induced and reinserted into the *lsr2* gene. Thereafter, a downstream *lsr2*‐silenced LOS synthesis gene island is activated and triggers abnormal PIM accumulation in the cell envelope of the *lsr2* deletion strain, which results in the phage absorption defect of the mycobacterium, defending against phage infection. Therefore, we uncovered a new signal pathway of IS transposition‐triggered antiphage immunity in *M. smegmatis*. Our study shows that endogenous ISs in mycobacteria can activate the defensive gene islands, thereby helping bacteria quickly acquire broad‐spectrum antiphage ability in a short period of time. This may represent a newly acquired defense strategy different from the previously reported CRISPR antiphage mechanism.

ISs exist widely in multiple sequenced genomes of mycobacteria, including *M. tuberculosis* ^29^ and *M. smegmatis* (https://www.ncbi.nlm.nih.gov/nuccore/CP000480.1), but their roles in the evolution of bacterial antiphage defense remain to be characterized. In the present study, when the genomes of all 32 isolated antiphage *M. smegmatis* strains were sequenced, an interesting finding is that IS*1096* transposition into the *lsr2* gene can occur at high frequencies, resulting in broad‐spectrum antiphage activity of the mutant mycobacterium. Lsr2 is a conserved nucleoid‐associated protein and widely exists in mycobacteria and other actinobacteria^31^. In *M. smegmatis* and *M. tuberculosis*, Lsr2 directly or indirectly controls the transcription of many genes^34^, ^35^, ^36^, ^37^. Very recently, the function of the *lsr2* gene has also been connected to the productive mycobacteriophage infection and Lsr2 was found to reorganize away from host replication foci and help establish zones of phage DNA replication^38^. However, the *lsr2* inactivation‐triggered regulating pathway for the antiphage phenotype remains largely unclear. In the current study, we characterized an Lsr2‐regulated downstream gene island, which is required for the antiphage K4JX5 defense of the *lsr2* deletion strain. This finding uncovered a new antiphage regulatory pathway activated by IS transposition under the pressure of phage infection in mycobacteria, which is clearly different in mechanism from the previous discovery^38^, and provided new insight into the natural evolution of bacterial antiphage defense.

Another interesting finding in the present study is that the LOS synthesis gene island triggers abnormal PIM accumulation in the cell envelope of the *lsr2* deletion strain, resulting in a phage absorption defect and *lsr2* inactivation‐triggered antiphage K4JX5 immunity. For phage absorption, bacterial receptors can be surface‐anchored protein^9^, extracellular or capsule polysaccharides^10^, ^11^, ^46^, and flagella^12^. Although over 12,000 mycobacteriophages have been described, to date, very few phage‐binding receptors have been clearly identified from their host mycobacteria. It has been proposed that *M. smegmatis* I3 uses glycopeptide lipids as receptors^42^, and Wetzel et al. suggested that the surface component trehalose polyphylenates of mycobacteria may be a potential receptor for phage BPs and Muddy^43^. A very recent study suggested that phage‐binding receptors in *M. smegmatis* are associated with the metabolic homeostasis of phospholipids^30^. Consistently, in the present study, when comparing differential lipid components between the wild‐type and Δ*lsr2* strains, the contents of eight different Ac_1_PIMs in the Δ*lsr2* strain significantly increased (Figure 5B), resulting in a phage absorption defect. This is most likely because additional Ac_1_PIMs were overproduced in the envelope of *lsr2* deletion mycobacterium to hinder subsequent receptor recognition by phage, but the exact mechanism for the phage absorption defect remains to be further clarified. Strikingly, when the complete LOS synthesis gene island or its single gene was co‐deleted from the Δ*lsr2* strain, the content differences of these Ac_1_PIMs in the recombinant strain were not noticeable and even disappeared (Figure 5). This is consistent with our observation that *lsr2* deletion resulted in a phage K4JX5 absorption defect and the complete LOS synthesis gene island is required for this defect phenotype. Further support for this point is that a mutant phage, K4JX5‐Mut, had been successfully evolved with good absorption capacity and could successfully escape from the defense of the *lsr2* deletion strain (Figure 7). Taken together, our results suggest that the function of the LOS synthesis gene island is closely related to antiphage defense of *M. smegmatis* and its precise regulation is essential for maintaining effective phage absorption.

In the present study, we have provided data to show that *lsr2* inactivation induced a broad‐spectrum resistance to multiple clusters of mycobacteriophages, including, but not limited to, clusters A3GX4, A4ZJ24, A22GX2, K3GX1, K4JX5, G1HA1, L1HA1, I1GX4, and F1GX13 (Figure 1B, middle panel), and we used K4JX5 as an example to identify the signal pathway and mechanism by which IS transposition activates antiphage immunity through the *lsr2*‐silenced LOS synthesis gene island. However, this does not mean that all the antiphage activities triggered by *lsr2* deletion in the mycobacterium are produced only through the LOS synthesis gene island, and it is most likely that the LOS synthesis gene island is only responsible for partial mechanisms. Consistently, although the co‐deleted strain, Δ*lsr2*‐Ko (*MSMEG_4727–4737*), re‐acquired sensitivity to some phages used in the experiment shown in Figure 2C (middle panel), it still retained partial resistance to some other phages such as K3GX1, F1GX13, and G1HA1. Therefore, our results suggest that the *lsr2* deletion in the mycobcterial strain may trigger diverse antiphage mechanisms which would be achieved through different pathways to different phages, which is consistent with a previous observation^38^. However, the specific mechanisms and signal pathways by which *lsr2* inactivation is triggered through uncharacterized downstream genes, ultimately causing immunity to different phages, remain unclear.

In conclusion, we successfully uncovered a new signaling pathway for activating *lsr2*‐silenced antiphage immunity by IS transposition in *M. smegmatis*. This work has greatly enriched our understanding of the evolution strategy and molecular mechanism of mycobacterial antiphage defense.

## MATERIALS AND METHODS

### Bacterial strains, phages, and cultivation conditions

Bacterial strains and phages used in this work are presented in Table S4. *Escherichia coli* strains were cultured in LB medium at 37°C, whereas *M. smegmatis* mc^2^ 155 strains were cultivated in 7H9 medium (BD Difco) containing 0.2% glycerol and 0.05% Tween 80 (Sigma‐Aldrich), or in 7H10 medium (BD Difco) containing 0.5% glycerol at 37°C. The corresponding antibiotic was added to the culture with shaking at 160 rpm if required. Mycobacteriophages were isolated from different soil sources in China, and the wild‐type *M. smegmatis* strain was used for their propagation.

### Construction of recombinant *M. smegmatis* strains

Deletions of the *lsr2* gene in the wild‐type *M. smegmatis* strain, or the genes including *MSMEG_4728*, *MSMEG_4731*, *MSMEG_4733*, *MSMEG_4734*, *MSMEG_4737*, *MSMEG_4727–4737*, *MSMEG_4727–4732*, *MSMEG_4728–4732*, and *MSMEG_4733–4737*, or the promoters such as S1 (*MSMEG_4727*p), S2 (*MSMEG_4735*p), and S3 (*MSMEG_4737*p) in the *lsr2* deletion strain, were performed according to previously described procedures^30^. In brief, a recombinant suicide plasmid was constructed and transferred into the corresponding mycobacterial strains according to the reported procedures. Finally, white colonies were selected and confirmed by PCR amplification.

For gene complementation, the pMV261 vector was used following previous procedures^47^. Briefly, the complete *MSMEG_4733–4737* gene island or each of DNA segments containing partial genes or regulator gene of the island was amplified by PCR, respectively. The amplified *lsr2* gene was inserted into the pMV261 vector between the *Bam*HI and *Hin*dIII restriction sites, while other amplicons were separately assembled into the vector between the *Xba*I and *Hin*dIII restriction sites using the cloning kit (Genesand). The resulting plasmids were transferred into the corresponding *M. smegmatis* strains and plated on 7H10 medium supplemented with 25 μg/ml kanamycin.

### Isolation of mycobacterial mutants with phage resistance

The wild‐type strain of *M. smegmatis* was grown in 7H9 medium to an OD_600_ of 1.0, followed by co‐incubation with phage TM4 at 30°C for 2 days. One milliliter of the culture was collected and resuspended in 7H9 medium. The cells were then diluted and plated on 7H10 medium until single colonies were generated. Subsequently, single colonies were picked and cultured in 7H9 medium to assay their phage resistance. The genome of the mutant strain was extracted and sequenced by BGI group.

### Protein expression and purification

The genes encoding Lsr2 and MSMEG_5860 proteins were amplified by PCR using their specific primers listed in Table S5. The amplicons were separately assembled into the pGEX‐6T‐1 and pET28a (Merck) vectors and then transformed into *E. coli* BL21 (DE3). Recombinant strains were cultured, induced, and treated using the methods described previously^48^. The eluted proteins were dialyzed in a low‐salt buffer (20 mM Tris‐HCl, 100 mM NaCl, 1 mM dl‐dithiothreitol, 10% glycerol) for 1 h and stored at −80°C. Protein concentration was determined using the Coomassie Brilliant Blue assay.

### Electrophoretic mobility shift assays

The DNA‐binding ability of His‐GST‐Lsr2 and His‐MSMEG_5860 was determined by EMSA, as described previously^49^. Briefly, the promoter DNA substrates were amplified by PCR using their primers (Table S5). The amplified DNA fragments were incubated with various amounts of protein at room temperature for 20 min and then electrophoresed according to previously described procedures^49^.

### CRISPR/dCas9 interference (CRISPRi) assays

CRISPRi assays were performed according to the procedures described previously^50^. Briefly, the small‐guide RNA (sgRNA), which consists of a 20 bp region near the 5′‐end of the non‐template strand preceding the protospacer‐adjacent motif (Table S6), was designed to inhibit gene transcription elongation by targeting the coding sequence. The sgRNA primers were annealed and inserted into the *Bsm*BI site of the pLJR962 vector (Addgene), which were subsequently transferred into *M. smegmatis* strains using the above‐mentioned method. When necessary, 200 ng/ml anhydrotetracycline hydrochloride (ATc) was added to inhibit the expression of the sgRNA‐targeted gene in the recombinant strain.

### Lipidomic assays

Lipidomic assays were performed with several modifications as described previously^30^. In brief, mycobacterial strains were grown in 7H9 medium and then harvested when an OD_600_ of 1.0 was reached. Subsequently, the whole‐lipidomic assays, including lipid extraction, ultra‐performance liquid chromatography, mass spectrometry, and lipid structural analysis were conducted by Novogene. Databases such as NIST (https://www.nist.gov/), Lipidmaps (http://www.lipidmaps.org), and Mtb LipidDB (https://www.ncbi.nlm.nih.gov/ pmc/articles/PMC3073466/) were used for mycobacterial metabolite identification. For each sample, six independent replicates were obtained, and the statistical significance (*p* value) and fold change of the metabolites between the means of the two groups were calculated by univariate analysis (*t*‐test).

### Quantitative proteomic (iTRAQ) analysis

Quantitative proteomic analysis was performed by Novogene as described^51^. Briefly, the wild‐type and *lsr2* deletion strains of *M. smegmatis* were grown in 7H9 medium to an OD_600_ of 1.0 and then collected. The cells were treated and analyzed according to previously described procedures^51^. The protein quantification results were statistically analyzed using a *t*‐test. Proteins with *p* < 0.05 and |log_2_FC| > 0.6 were defined as differentially expressed proteins.

### qRT‐PCR analysis

Recombinant *M. smegmatis* strains were cultivated in 7H9 medium to an OD_600_ of 1.0 and then harvested. RNA extraction, reverse‐transcription reaction, and qRT‐PCR assays were conducted as described previously^52^. The expression levels of all genes were normalized to the level of the *sigA* gene. Relative quantification of gene expression level was performed using the $2^{-∆∆\mathrm{CT}}$ method.

### Assays for mycobacterial resistance to phage

Phage susceptibility profiles of *M. smegmatis* strains were evaluated using standard plaque assays^30^. Mycobacteriophage was serially diluted and vortexed. Subsequently, 2 µl of the diluted phages were dropped onto mycobacterial cell lawns (OD_600_ = 1.0) and incubated at 37°C for 24 or 48 h until plaque formation. Additionally, phage titer assays were conducted. Briefly, 100 μl of the diluted phages were mixed with 1 ml cultures of mycobacterial strain and 5 ml of LB‐agar medium. The mixture was then plated on 7H10 medium at 37°C. After 48 h incubation, the number of plaques was counted. The phage resistance of the gene‐silenced strain was evaluated with and without 200 ng/ml of ATc using the described procedures.

### Phage adsorption assays

Phage adsorption assays were conducted as described earlier^30^. Briefly, *M. smegmatis* strains were cultivated in 7H9 medium containing 10% OADC, and then collected when an OD_600_ of 1.0 was reached. The cell pellets were treated according to the previously reported procedures^30^. Subsequently, K4JX5 phage (MOI = 0.01) was mixed with three 5 ml cultures of each strain. At the indicated time points, 1 ml aliquots were removed and centrifuged. The phage number in the supernatant was determined by infecting the wild‐type *M. smegmatis* strain on 7H10 medium.

### DNA electroporation assay

The genome DNA of K4JX5 Phage was extracted using the phenol‐chloroform method as described earlier^53^. A total of 200 ng of the genomic DNA was electroporated into *M. smegmatis* strains and supplemented with 900 μl 7H9 medium, followed by shaking at 160 rpm at 37°C for 1 h. The cells were collected, re‐suspended with 200 μl of 7H9 medium, and then mixed with 1 ml of the wild‐type *M. smegmatis* strain and 5 ml of LB‐agar medium. The mixture was plated on 7H10 medium and incubated at 37°C. After 48 h incubation, the plaque number was counted.

### Isolation of K4JX5 mutants

The diluted K4JX5 phage solution was dropped onto the lawn of the *lsr2* deletion strain of *M. smegmatis* until clear plaques were observed. The plaque was picked and incubated with the knockout strain at 37°C. After 2 days of incubation, 1 ml of the culture was removed and centrifuged. The supernatant was mixed with 1 ml of the *lsr2* deletion strain in 5 ml of LB‐agar medium and incubated on 7H10 medium at 37°C for 48 h. Single plaques were picked and enriched, followed by genome sequencing.

### Statistical analysis

Data are presented as mean ± SD, *n* = 3–6. All statistical analyses were performed using GraphPad Prism 8, and significance was calculated and listed in the figure legend.

## AUTHOR CONTRIBUTIONS

**Yakun Li**: Data curation (lead); formal analysis (lead). **Yuyun Wei**: Formal analysis (supporting); investigation (supporting); methodology (supporting). **Xiao Guo**: Data curation (supporting); formal analysis (supporting); software (equal). **Xiaohui Li**: Data curation (supporting); methodology (supporting); software (supporting); visualization (equal). **Lining Lu**: Project administration (supporting). **Lihua Hu**: Project administration (supporting); resources (supporting). **Zheng‐Guo He**: Conceptualization (equal); data curation (equal); formal analysis (equal); funding acquisition (lead); investigation (equal); methodology (lead); project administration (lead); resources (lead); supervision (lead); writing—original draft (lead); writing—review and editing (lead).

## ETHICS STATEMENT

No animals or humans were involved in this study.

## CONFLICT OF INTERESTS

The authors declare no conflict of interests.

## Supporting information

Supporting information.

Supporting information.

Supporting information.

Supporting information.

Supporting information.

Supporting information.

Supporting information.

## Data Availability

Lipidomic data are available on Zenodo: 10.5281/zenodo.10506317.
