# Supplementary material for: Insertion sequence transposition activates antimycobacteriophage immunity through an lsr2‐silenced lipid metabolism gene island
Source: mLife. 2024 Mar 26;3(1):87–100. doi: 10.1002/mlf2.12106 (PMC11139207; doi:10.1002/mlf2.12106)
Supplement: Supplementary file 1 — Supporting information. [file MLF2-3-87-s005.docx]

**Supplemental information**

**Insertion sequence transposition activates anti-mycobacteriophage immunity through a *lsr2*-silenced lipid metabolism gene island**

**Yakun Li^1^, Yuyun Wei^1^, Xiao Guo^1^, Xiaohui Li^1^, Lining Lu,^1^ Lihua Hu,^1^ Zheng-Guo He*****^,1^**

^1^State Key Laboratory for Conservation and Utilization of Subtropical Agro-bioresources, Guangxi Research Center for Microbial and Enzyme Engineering Technology, College of Life Science and Technology, Guangxi University, Nanning 530004, China.

*To whom correspondence should be addressed: College of Life Science and Technology, Guangxi University, Nanning 530004, China.

Email: [hezhengguo2019@163.com](mailto:hezhengguo2019@163.com)

Tel: +86-771-3225146, Fax: +86-771-3225146

**Key words:** Insertion sequence; bacteriophage; gene island; anti-phage defense; mycobacteria

**Running title:** Insertion sequence activates mycobacterial anti-phage immunity


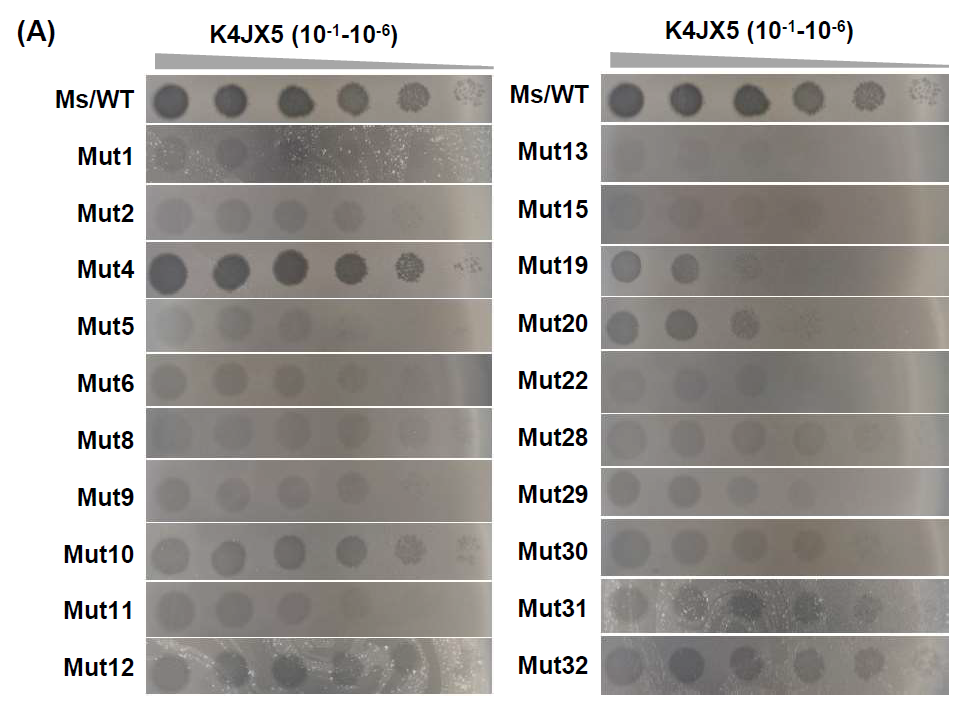


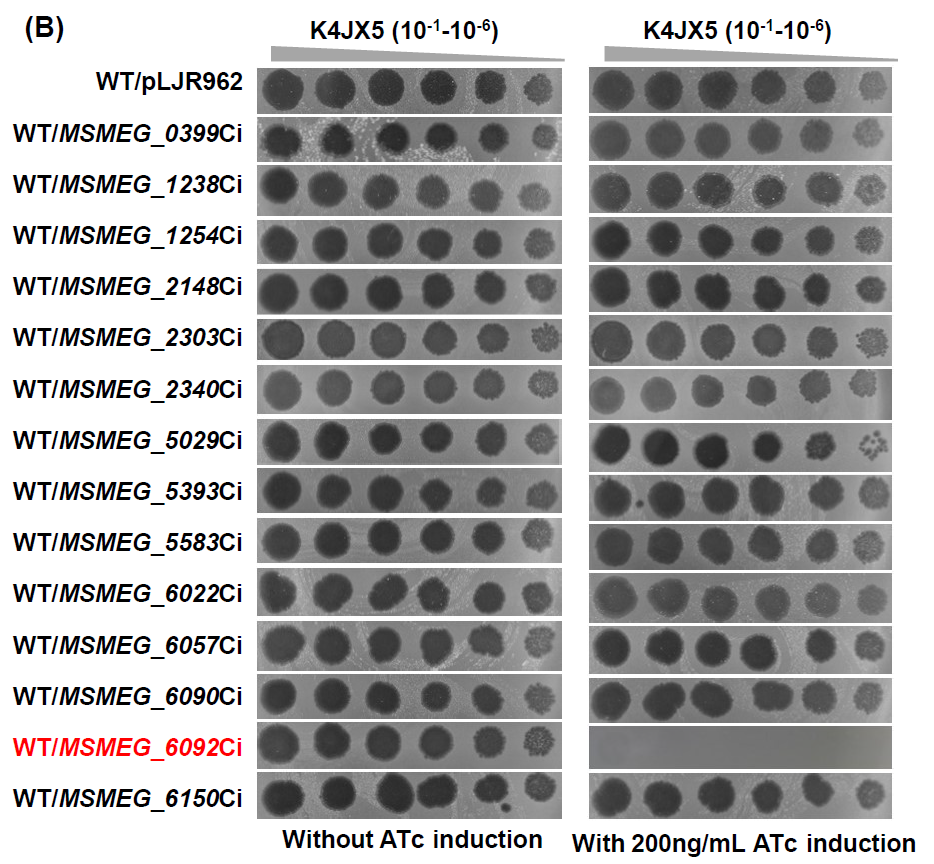


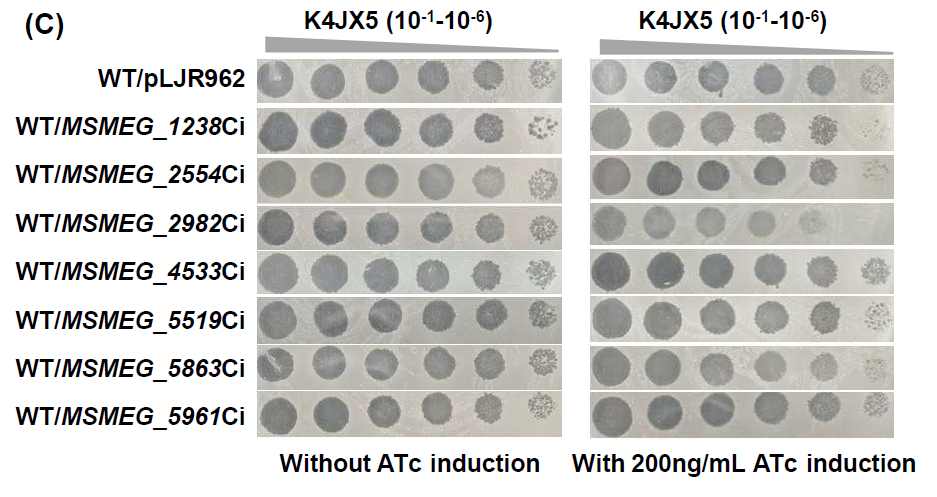


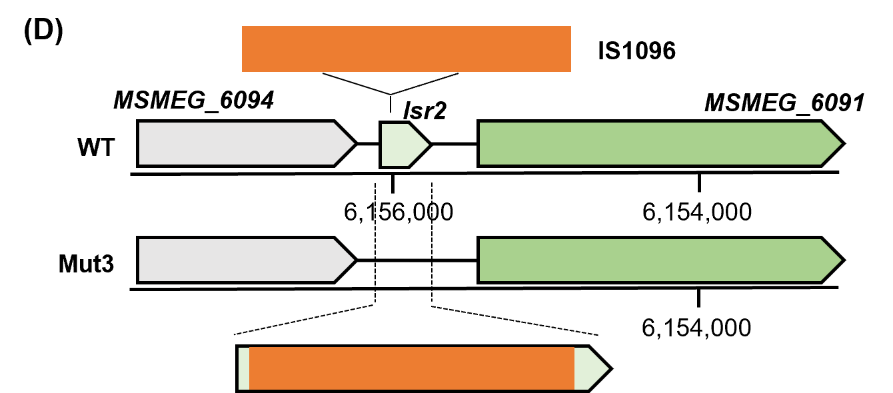


**Figure S1. IS1096 transposition into the *lsr2* gene of *M. smegmatis* induces a broad anti-phage phenotype.**

(**A**) Plaque formation ability of phage K4JX5 on plates containing 20 different mutant strains of *M. smegmatis,* in which genome contains a IS element. The K4JX5 dilution indicated at the top was spotted on a lawn of the *M. smegmatis* strain. Mut1-32 represent mutant strains formed by different types of insertion sequences in the genome of *M. smegmatis.* (**B**) CRISPRi assays for the effects of silencing expression of genes induced by TM4 insertion in the mutant strains on plaque-forming ability of phage K4JX5. WT/*MSMEG_0399*Ci, WT/*MSMEG_1238*Ci, WT/*MSMEG_1254*Ci, WT/*MSMEG_2148*Ci, WT/*MSMEG_5029*Ci, WT/*MSMEG_5393*Ci, WT/*MSMEG_5583*Ci, WT/*MSMEG_6022*Ci, WT/*MSMEG_6057*Ci, WT/*MSMEG_6090*Ci, WT/*MSMEG_6092*Ci, and WT/*MSMEG_6150*Ci represent recombinant strains containing the corresponding gene, which expression is repressed by 200 ng/mL ATc. (**C**) CRISPRi assays for the effects of silencing expression of genes induced by ISs in the mutant strains on plaque-forming ability of phage K4JX5. WT/MSMEG_2554Ci, WT/MSMEG_2982Ci, WT/MSMEG_4533Ci, WT/MSMEG_5519Ci, WT/MSMEG_5863Ci, WT/MSMEG_5961Ci, represent recombinant strains containing the corresponding gene, which expression is repressed by induction of 200 ng/mL ATc.(**D**) Schematic representation of the *lsr2* (*MSMEG_6092*) locus and its mutant with the insertion site of the IS1096 transposon.

**
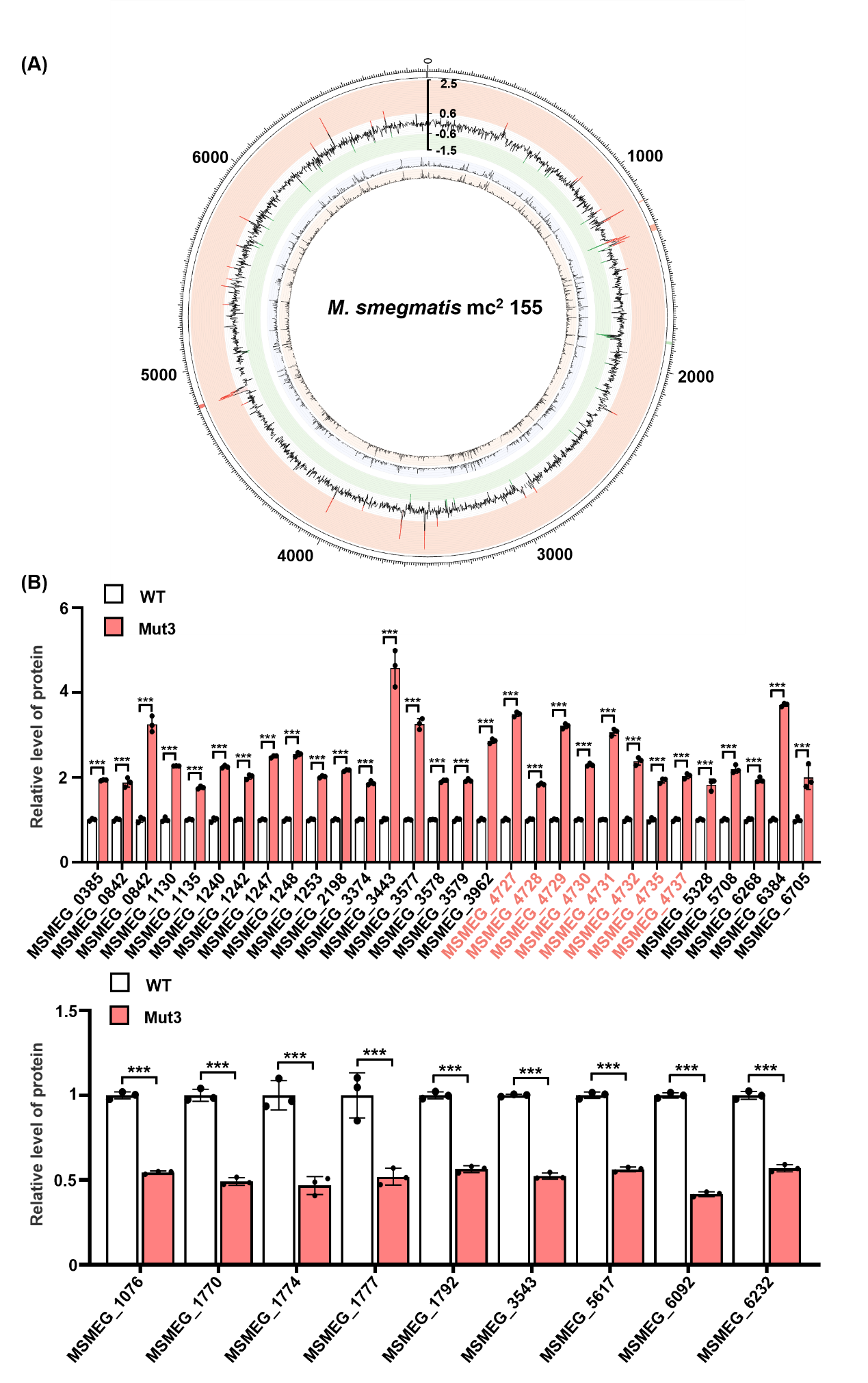
**

**Figure S2. Proteomic assays for the effect of the *lsr2* deletion on global gene expression of *M. smegmatis*.**

(**A**) Circos plot showing global changes of protein expression levels in *lsr2* insertion mutant *M. smegmatis* mc^2^ 155 if compared to that in the wild type strain. The line in the internal track shows the baseline, the green regions represent 33 down-regulated proteins (log_2_FC < -0.6 and *P*-value < 0.05) and the red regions represent 48 up-regulated proteins (log_2_FC > 0.6 and *P*-value < 0.05). The most-outer track represents the genome of the *lsr2* insertion mutant strain. The inner and most-inner blue and orange circles correspond to the expression of each gene (represented as intensity values) in *lsr2* insertion mutant and WT strain, respectively. In both of the colored circles, thin gray circular lines represent an intensity value of 10,000, with a limit at 40,000. (**B**) Upper panel, 30 up-regulated genes (log_2_ FC > 0.8 and *P*-value < 0.05) in the Δ*lsr2* strain compared to wild-type strain, which covered 8 genes of the LOS island indicated by pink print. Lower panel, 9 down-regulated genes (log_2_ FC < -0.8 and *P*-value < 0.05) in the *lsr2* insertion mutant strain. Error bars represent the SD from three replicates. Statistical analysis was conducted by unpaired two-tailed Student’s t test (****P*-value < 0.001).


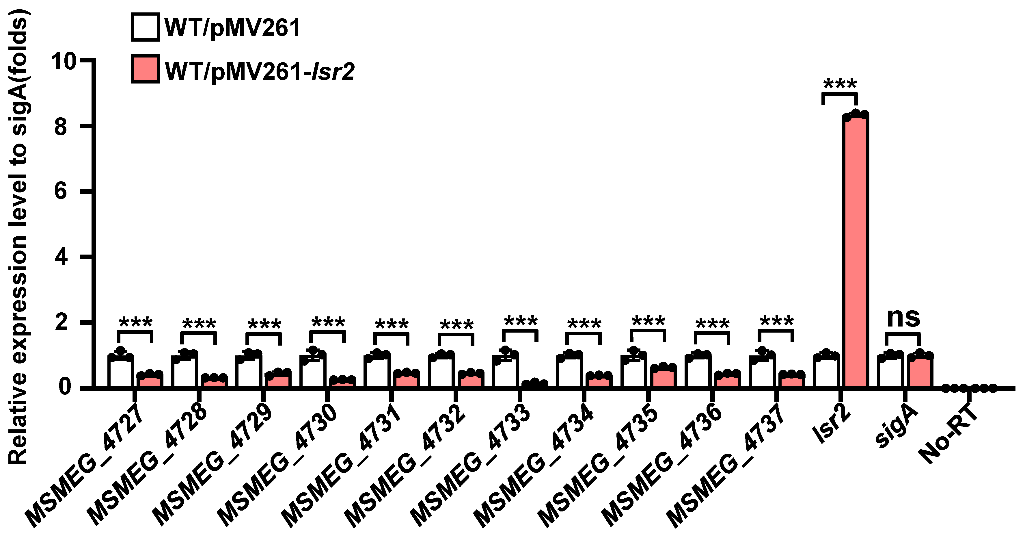


**Figure S3. Lsr2 inhibits the expression of the LOS island genes**

Quantitative real-time PCR assays for the differential expression of the LOS island genes in wildtype and *lsr2*-deleted *M. smegmatis* strains. *sigA* was used as a control. Error bars represent the SD from three replicates. Statistical analysis was conducted by unpaired two-tailed Student’s t test (****P*-value < 0.001).


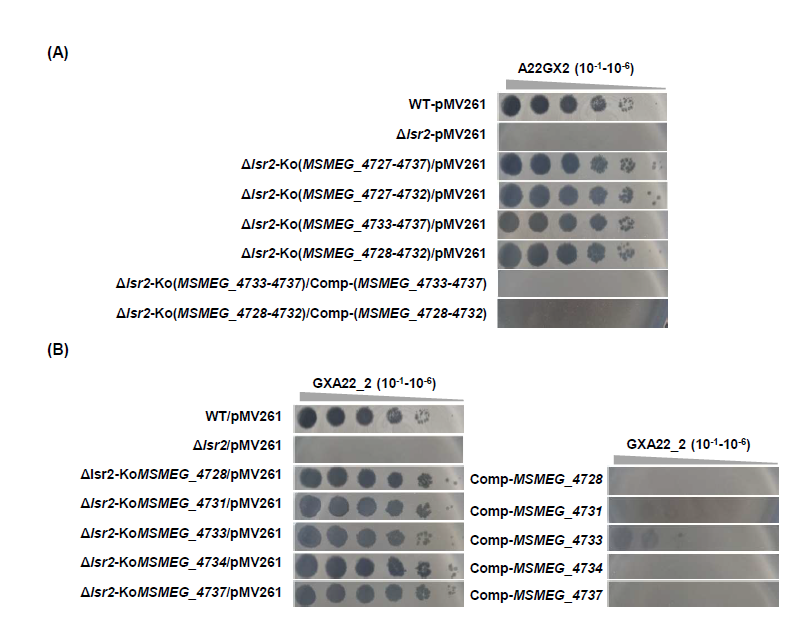


**Figure S4. Most of LOS island genes are required for the *lsr2* inactivation- triggered pathway to fight against phage A22GX2**

(**A**) Effects of the complete or partial deletion of the LOS gene island on the resistance of the *lsr2*-deleted strain to phage A22GX2. Left, the diagram of deleting the LOS island or partial genes. Right, plaque formation ability of phage K4JX5 on plates containing different recombinant *M. smegmatis* strains which is same as shown in Figure 4A.(**B**) Effects of co-deleting a single gene of the LOS island in Δ*lsr2* strain on the plaque formation of phage A22GX2 by spotting phage on the lawns of different recombinant *M. smegmatis* strains which is same as shown in Figure 4C.


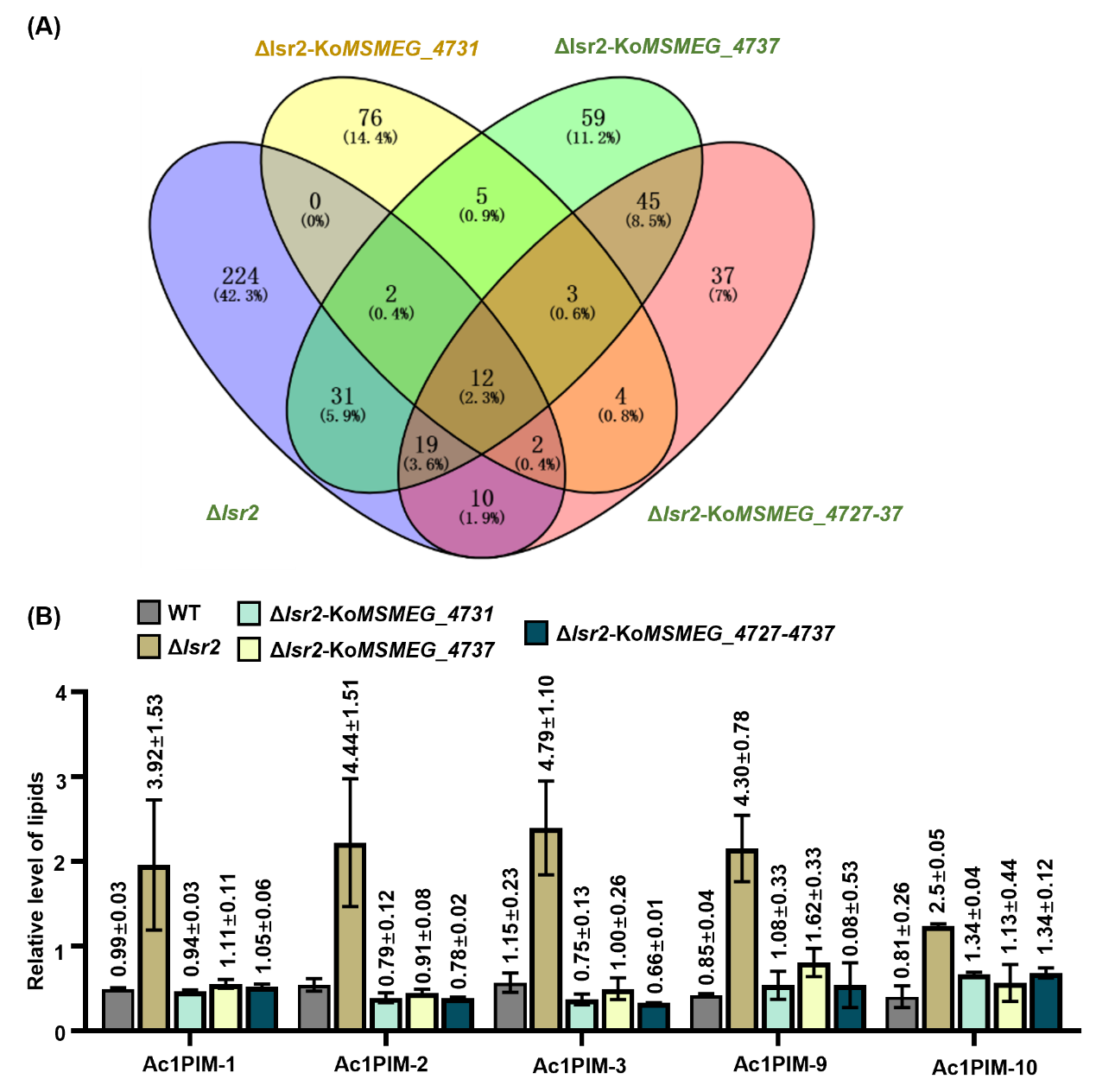


**Figure S5. Effects of the LOS island genes expression on the morphology and intracellular lipid levels of the *lsr2*-deleted strain**

(**A**) Intersection of the differentially expressed lipids (fold change ≥ 1.5 and *P* < 0.05) among Δ*lsr2*, Δ*lsr2*-Ko*MSMEG_4731*, Δ*lsr2*-Ko*MSMEG_4737*, and Δ*lsr2*-Ko*MSMEG_4727-4737.*(**B**) Column graphs showing the relative levels of 5 Ac1PIM lipids in the above-mentioned strains determined by lipidomic assays. Ac1PIM-1, Ac1PIM-3, Ac1PIM-9, and Ac1PIM-10 represents lipids as shown in Figure 5B.

**
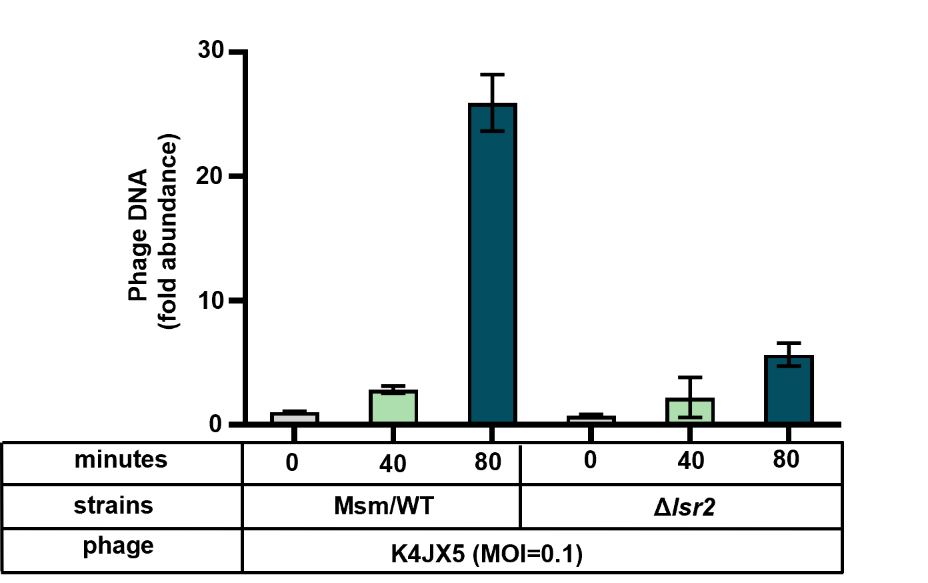
**

**Figure S6. Deletion of *lsr2* impairs the replication ability of phage K4JX5.**

Quantitative real-time PCR was utilized to determine the relative abundance of phage DNA at various time points following phage K4JX5 infection. Error bars represent the SD from three replicates. Statistical analysis was conducted by unpaired two-tailed Student’s *t*-test (****P*-value <0.001).
