# Supplementary material for: Insertion sequence transposition activates antimycobacteriophage immunity through an lsr2‐silenced lipid metabolism gene island
Source: mLife. 2024 Mar 26;3(1):87–100. doi: 10.1002/mlf2.12106 (PMC11139207; doi:10.1002/mlf2.12106)
Supplement: Supplementary file 2 — Supporting information. [file MLF2-3-87-s002.docx]

**Table S1. List of TM4 transposon insertion position.**

| **Mutant** | **Insertion Gene** | **Insertion genome position** |
| --- | --- | --- |
| Mut1 | *MSMEG_6431* | 6498632 |
| Mut2 | *MSMEG_1237-MSMEG_1238* Spacer | 1307964 |
| Mut3 | *MSMEG_1816-MSMEG_1818* Spacer | 1892722 |
| Mut4 | *MSMEG_3991* | 4063412 |
| Mut5 | *MSMEG_5519* | 5607992 |
| Mut6 | *MSMEG_6547* | 6603948 |
| Mut7 | *MSMEG_5961* | 6022754 |
| Mut8 | *MSMEG_2554* | 2638852 |
| Mut9 | *MSMEG_1238* | 1309255 |
| Mut10 | *MSMEG_6248* | 6313661 |
| Mut11 | *MSMEG_5863* | 5929328 |
| Mut12 | *MSMEG_6431* | 6498645 |
| Mut13 | *MSMEG_6431* | 6498645 |
| Mut14 | *MSMEG_4533* | 4620693 |
| Mut15 | *MSMEG_4929* | 5025705 |
| Mut16 | *MSMEG_2554* | 2638852 |
| Mut17 | *MSMEG_2554* | 2638852 |
| Mut18 | *MSMEG_2554* | 2638852 |
| Mut19 | *MSMEG_0092* | 117480 |
| Mut20 | *MSMEG_0092* | 117480 |
| Mut21 | *MSMEG_2554* | 2638852 |
| Mut22 | *MSMEG_2554* | 2638852 |
| Mut23 | *MSMEG_2554* | 2638852 |
| Mut24 | *MSMEG_2554* | 2638852 |
| Mut25 | *MSMEG_2554* | 2638852 |
| Mut26 | *MSMEG_2554* | 2638852 |
| Mut27 | *MSMEG_2554* | 2638852 |
| Mut28 | *MSMEG_2554* | 2638852 |
| Mut29 | *MSMEG_1238* | 1308410 |
| Mut30 | *MSMEG_2982* | 3047585 |
| Mut31 | *MSMEG_2537* | 2622610 |
| Mut32 | *MSMEG_0848* | 933654 |
