# Supplementary material for: Insertion sequence transposition activates antimycobacteriophage immunity through an lsr2‐silenced lipid metabolism gene island
Source: mLife. 2024 Mar 26;3(1):87–100. doi: 10.1002/mlf2.12106 (PMC11139207; doi:10.1002/mlf2.12106)
Supplement: Supplementary file 3 — Supporting information. [file MLF2-3-87-s007.docx]

**Table S2. IS transposon insertion position.**

| **Insertion Gene** | **Mutant** | **Transposon type** |
| --- | --- | --- |
| MSMEG_0399 | Mut1-t32 | IS1096 |
| MSMEG_1238 | Mut1-32 | IS1096 |
| MSMEG_1254 | Mut1-32 | IS6120 |
| MSMEG_2148 | Mut13, Mut14-32 | Unknown |
| MSMEG_2303 | Mut6 | Unknown |
| MSMEG_2340 | Mut7 | IS1096 |
| MSMEG_2823-25 | Mut2, Mut3, Mut6, Mut-32 | IS1549 |
| MSMEG_5029 | Mut19 | ISMsm1 |
| MSMEG_5181 | Mut1-32 | IS1549 |
| MSMEG_5393 | Mut9 | ISMsm1 |
| MSMEG_5583 | Mut1-32 | ISMsm1 |
| MSMEG_6022 | Mut11 | ISMsm16162 |
| MSMEG_6057 | Mut1 | ISMsm1 |
| MSMEG_6090-MSMEG_6091Spacer | Mut32 | IS1096 |
| MSMEG_6092 | Mut5, Mut7-9, Mut11, Mut14, Mut16-18, Mut21-30 | IS1096 |
| MSMEG_6148 | Mut11, Mut16, Mut30, Mut32 | IS1096 |
| MSMEG_6149-MSMEG_6150 Spacer | Mut1-10, Mut12-15, Mut17-29. Mut31 | IS1096 |
