# Supplementary material for: Insertion sequence transposition activates antimycobacteriophage immunity through an lsr2‐silenced lipid metabolism gene island
Source: mLife. 2024 Mar 26;3(1):87–100. doi: 10.1002/mlf2.12106 (PMC11139207; doi:10.1002/mlf2.12106)
Supplement: Supplementary file 4 — Supporting information. [file MLF2-3-87-s006.docx]

**Table S3. The expression changes of *MSMEG_4727-4737* in Δ*lsr2* strain compared to WT.**

| **Gene** | **Annotation** | **Fold Change** |
| --- | --- | --- |
| *MSMEG_4727* | mycocerosic acid synthase | 3.2 |
| *MSMEG_4728* | condensation domain-containing protein | 3.4 |
| *MSMEG_4729* | hypothetical protein | 3.8 |
| *MSMEG_4730* | hypothetical protein | 4 |
| *MSMEG_4731* | acyl-CoA synthetase | 3.7 |
| *MSMEG_4732* | glycosyl transferase family protein | 3.3 |
| *MSMEG_4733* | hypothetical protein | 2.2 |
| *MSMEG_4734* | hypothetical protein | 1.6 |

**References**

Kołodziej M, Łebkowski T, Płociński P, Hołówka J, Paściak M, Wojtaś B, et al. Lsr2 and Its Novel Paralogue Mediate the Adjustment of Mycobacterium smegmatis to Unfavorable Environmental Conditions. mSphere 2021:6:e00290-21.
