# Supplementary material for: Insertion sequence transposition activates antimycobacteriophage immunity through an lsr2‐silenced lipid metabolism gene island
Source: mLife. 2024 Mar 26;3(1):87–100. doi: 10.1002/mlf2.12106 (PMC11139207; doi:10.1002/mlf2.12106)
Supplement: Supplementary file 5 — Supporting information. [file MLF2-3-87-s004.docx]

**Table S4. Strains, phages and plasmids used in this study.**

| **Plasmid, phage or Strain** | **Relevant genotype or feature** | **Source or reference** |
| --- | --- | --- |
| Plasmid |  |  |
| pET28a | Kanr, T7 lac promoter, N-terminal His_6_ | Novagen |
| pET -*MSMEG_5860* | *MSMEG_5860* inserted in *EcoR*I*-Xba*I of pET28a | This study |
| pGEX-6t-1 | Amp^r^, *lacZ* operon, Tac promotor, His-GST-Tag | Previous study^[S1]^ |
| pGEX-6t-*lsr2* | *lsr2* inserted in *BamH*I*-Xho*I of pGEX-6t-1 | This study |
| pLJR962 | Kan^r^, Sth1 sgRNA scaffold and *dCas9*, TetR, L5 attP and Int for *M. smegmatis* | Addgene |
| pLJR962-*MSMEG_0399* | *MSMEG_1192* sgRNA inserted in *BsmB*I of pLJR962 | This study |
| pLJR962-*MSMEG_1238* | *MSMEG_1238* sgRNA inserted in *BsmB*I of pLJR962 | This study |
| pLJR962-*MSMEG_1254* | *MSMEG_1254* sgRNA inserted in *BsmB*I of pLJR962 | This study |
| pLJR962-*MSMEG_2148* | *MSMEG_2148* sgRNA inserted in *BsmB*I of pLJR962 | This study |
| pLJR962-*MSMEG_2303* | *MSMEG_2303* sgRNA inserted in *BsmB*I of pLJR962 | This study |
| pLJR962-*MSMEG_2430* | *MSMEG_2430* sgRNA inserted in *BsmB*I of pLJR962 | This study |
| pLJR962-*MSMEG_5029* | *MSMEG_5029* sgRNA inserted in *BsmB*I of pLJR962 | This study |
| pLJR962-*MSMEG_5393* | *MSMEG_5393* sgRNA inserted in *BsmB*I of pLJR962 | This study |
| pLJR962-*MSMEG_5583* | *MSMEG_5583* sgRNA inserted in *BsmB*I of pLJR962 | This study |
| pLJR962-*MSMEG_6022* | *MSMEG_6022* sgRNA inserted in *BsmB*I of pLJR962 | This study |
| pLJR962-*MSMEG_6057* | *MSMEG_6057* sgRNA inserted in *BsmB*I of pLJR962 | This study |
| pLJR962-*MSMEG_6090* | *MSMEG_6090* sgRNA inserted in *BsmB*I of pLJR962 | This study |
| pLJR962-*MSMEG_6092* | *MSMEG_6092* sgRNA inserted in *BsmB*I of pLJR962 | This study |
| pLJR962-*MSMEG_6150* | *MSMEG_6150* sgRNA inserted in *BsmB*I of pLJR962 | This study |
| pLJR962-*MSMEG_4727* | *MSMEG_4727* sgRNA inserted in *BsmB*I of pLJR962 | This study |
| pLJR962-*MSMEG_4728* | *MSMEG_4728* sgRNA inserted in *BsmB*I of pLJR962 | This study |
| pLJR962-*MSMEG_4729* | *MSMEG_4729* sgRNA inserted in *BsmB*I of pLJR962 | This study |
| pLJR962-*MSMEG_4730* | *MSMEG_4730* sgRNA inserted in *BsmB*I of pLJR962 | This study |
| pLJR962-*MSMEG_4731* | *MSMEG_4731* sgRNA inserted in *BsmB*I of pLJR962 | This study |
| pLJR962-*MSMEG_4732* | *MSMEG_4732* sgRNA inserted in *BsmB*I of pLJR962 | This study |
| pLJR962-*MSMEG_4735* | *MSMEG_4735* sgRNA inserted in *BsmB*I of pLJR962 | This study |
| pLJR962-*MSMEG_4737* | *MSMEG_4737* sgRNA inserted in *BsmB*I of pLJR962 | This study |
| pMV261 | Kan^r^, pAL5000 replicon | Previous study^[S2]^ |
| pMV261-*MSMEG_6092* | *MSMEG_6092* in *Not*I-*Xba*I sites of pMV261 | This study |
| pMV261-*MSMEG_4728-4732* | *MSMEG_4728-4732* in *Xba*I-*Hind*Ⅲ sites of pMV261 | This study |
| pMV261-*MSMEG_4733-4737* | *MSMEG_4733-4737* in *Xba*I-*Hind*Ⅲ sites of pMV261 | This study |
| pMV261-*MSMEG_4728* | *MSMEG_4728 inserted in EcoRI-XbaI of pMV261* | This study |

| ***Continued*** |  |  |
| --- | --- | --- |
| **Plasmid, phage or Strain** | **Relevant genotype or feature** | **Source or reference** |
| pMV261-*MSMEG_4731* | *MSMEG_4731* inserted in *EcoRI-XbaI* of pMV261 | This study |
| pMV261-*MSMEG_4733* | *MSMEG_4733* inserted in *BamH*Ⅰ-*Hind*Ⅲ of pMV261 | This study |
| pMV261-*MSMEG_4734* | *MSMEG_4734* inserted in *Bam*HⅠ-*Hind*Ⅲ of pMV261 | This study |
| pMV261-*MSMEG_4737* | *MSMEG_4737* inserted in *EcoR*I-*Xba*I of pMV261 | This study |
| Strain |  |  |
| *E. coli* BL21 (DE3) |  | TaKaRa |
| *M. smegmatis* MC^2^ 155 |  | ATCC |
| Ms/WT | *M. smegmatis* MC^2^ 155 | ATCC |
| Δ*lsr2* | *M. smegmatis* MC^2^ 155 with *MSMEG_6092* deletion | This study |
| Δ*lsr2-*Ko (*MSMEG_4727-4737*) | Δ*lsr2* with *MSMEG_4727-4737* deletion | This study |
| Δ*lsr2-*Ko (*MSMEG_4728-4732*) | Δ*lsr2* with *MSMEG_4728-4732* deletion | This study |
| Δ*lsr2-*Ko (*MSMEG_4733-4737*) | Δ*lsr2* with *MSMEG_4733-4737* deletion | This study |
| Δ*lsr2-*Ko*MSMEG_4728* | Δ*lsr2* with *MSMEG_4728* deletion | This study |
| Δ*lsr2-*Ko*MSMEG_4731* | Δ*lsr2* with *MSMEG_4731* deletion | This study |
| Δ*lsr2-*Ko*MSMEG_4733* | Δ*lsr2* with *MSMEG_4733* deletion | This study |
| Δ*lsr2-*Ko*MSMEG_4734* | Δ*lsr2* with *MSMEG_4734* deletion | This study |
| Δ*lsr2-*Ko*MSMEG_4737* | Δ*lsr2* with *MSMEG_4737* deletion | This study |
| Δ*lsr2-*KoSⅠ | Δ*lsr2* with SⅠ deletion | This study |
| Δ*lsr2-*KoSⅡ | Δ*lsr2* with SⅡ deletion | This study |
| Δ*lsr2-*KoSⅢ | Δ*lsr2* with SⅢ deletion | This study |
| WT*/*pMV261 | *M. smegmatis* MC2 155 with pMV261 | This study |
| WT*/*pMV261-*lsr2* | *M. smegmatis* MC2 155 with pMV261-*MSMEG_6092* | This study |
| Δ*lsr2/*pMV261 | Δ*lsr2* with pMV261 | This study |
| Δ*lsr2/*Comp-*lsr2* | Δ*lsr2* with pMV261-*MSMEG_6092* | This study |
| Δ*lsr2-*Ko(*MSMEG_4727-4737*)*/*pMV261 | Δ*lsr2-*Ko(*MSMEG_4727-4737*) with pMV261 | This study |
| Δ*lsr2-*Ko (*MSMEG_4728-4732*)*/*pMV261 | Δ*lsr2-*Ko(*MSMEG_4728-4732*) with pMV261 | This study |
| Δ*lsr2-*Ko(*MSMEG_4733-4737*)*/*pMV261 | Δ*lsr2-*Ko(*MSMEG_4733-4737*) with pMV261 | This study |
| Δ*lsr2*-Ko(*MSMEG_4733-4737*)/Comp(*MSMEG_4733-4737)* | Δlsr2-Ko(*MSMEG_4733-4737*) with pMV26-  *MSMEG_4733-4737* | This study |

| ***Continued*** |  |  |
| --- | --- | --- |
| **Plasmid, phage or**  **Strain** | **Relevant genotype or feature** | **Source or reference** |
| Δ*lsr2-*Ko*MSMEG_4728*/pMV261 | Δ*lsr2-*Ko*MSMEG_4728* with pMV261 | This study |
| Δ*lsr2-*Ko*MSMEG_4731*/pMV261 | Δ*lsr2-*Ko*MSMEG_4731* with pMV261 | This study |
| Δ*lsr2-*Ko*MSMEG_4733*/pMV261 | Δ*lsr2-*Ko*MSMEG_4733* with pMV261 | This study |
| Δ*lsr2-*Ko*MSMEG_4734*/pMV261 | Δ*lsr2-*Ko*MSMEG_4734* with pMV261 | This study |
| Δ*lsr2-*Ko*MSMEG_4737*/pMV261 | Δ*lsr2-*Ko*MSMEG_4737* with pMV261 | This study |
| Comp-*MSMEG-4728* | Δ*lsr2-*Ko*MSMEG_4728* with pMV261-*MSMEG_4728* | This study |
| Comp-*MSMEG-4731* | Δ*lsr2-*Ko*MSMEG_4731* with pMV261-*MSMEG_4731* | This study |
| Comp-*MSMEG-4733* | Δ*lsr2-*Ko*MSMEG_4733* with pMV261-*MSMEG_4733* | This study |
| Comp-*MSMEG-4734* | Δ*lsr2-*Ko*MSMEG_4734* with pMV261-*MSMEG_4734* | This study |
| Comp-*MSMEG-4737* | Δ*lsr2-*Ko*MSMEG_4737* with pMV261-*MSMEG_4734* | This study |
| WT/pLJR962 | *M. smegmatis* MC^2^ 155 with pLJR962 | This study |
| Δ*lsr2*/pLJR962 | Δ*lsr2* with pLJR962 | This study |
| WT/*MSMEG_0399*Ci | *M. smegmatis* MC^2^ 155 with pLJR962-*MSMEG_0399* | This study |
| WT/*MSMEG_1238*Ci | *M. smegmatis* MC^2^ 155 with pLJR962-*MSMEG_1238* | This study |
| WT/*MSMEG_1254*Ci | *M. smegmatis* MC^2^ 155 with pLJR962-*MSMEG_1254* | This study |
| WT/*MSMEG_2148*Ci | *M. smegmatis* MC^2^ 155 with pLJR962-*MSMEG_2148* | This study |
| WT/*MSMEG_2303*Ci | *M. smegmatis* MC^2^ 155 with pLJR962-*MSMEG_2303* | This study |
| WT/*MSMEG_2340*Ci | *M. smegmatis* MC^2^ 155 with pLJR962-*MSMEG_2340* | This study |
| WT/*MSMEG_5029*Ci | *M. smegmatis* MC^2^ 155 with pLJR962-*MSMEG_5029* | This study |
| WT/*MSMEG_5393*Ci | *M. smegmatis* MC^2^ 155 with pLJR962-*MSMEG_5393* | This study |
| WT/*MSMEG_5583*Ci | *M. smegmatis* MC^2^ 155 with pLJR962-*MSMEG_5583* | This study |
| WT/*MSMEG_6022*Ci | *M. smegmatis* MC^2^ 155 with pLJR962-*MSMEG_6022* | This study |
| WT/*MSMEG_6057*Ci | *M. smegmatis* MC^2^ 155 with pLJR962-*MSMEG_6057* | This study |
| WT/*MSMEG_6090*Ci | *M. smegmatis* MC^2^ 155 with pLJR962-*MSMEG_6090* | This study |
| WT/*MSMEG_6092*Ci | *M. smegmatis* MC^2^ 155 with pLJR962-*MSMEG_6092* | This study |
| WT/*MSMEG_6150*Ci | *M. smegmatis* MC^2^ 155 with pLJR962-*MSMEG_6150* | This study |
| WT/*MSMEG_2554*Ci  WT/*MSMEG_2982*Ci  WT/*MSMEG_4533*Ci | *M. smegmatis* MC^2^ 155 with pLJR962-*MSMEG_2554*  *M. smegmatis* MC^2^ 155 with pLJR962-*MSMEG_2982*  *M. smegmatis* MC^2^ 155 with pLJR962-*MSMEG_4533* | This study  This study  This study |
| WT/*MSMEG_5519*Ci | *M. smegmatis* MC^2^ 155 with pLJR962-*MSMEG_5519* | This study |
| WT/*MSMEG_5863*Ci | *M. smegmatis* MC^2^ 155 with pLJR962-*MSMEG_5863* | This study |
| WT/*MSMEG_5961*Ci | *M. smegmatis* MC^2^ 155 with pLJR962-*MSMEG_5961* | This study |
| Δ*lsr2*/*MSMEG_4727*Ci | Δ*lsr2* with pLJR962-*MSMEG_4727* | This study |
| Δ*lsr2*/*MSMEG_4728*Ci | Δ*lsr2* with pLJR962-*MSMEG_4728* | This study |
| Δ*lsr2*/*MSMEG_47290*Ci | Δ*lsr2* with pLJR962-*MSMEG_4729* | This study |
| Δ*lsr2*/*MSMEG_4730*Ci | Δ*lsr2* with pLJR962-*MSMEG_4730* | This study |
| Δ*lsr2*/*MSMEG_4731*Ci | Δ*lsr2* with pLJR962-*MSMEG_4731* | This study |

| ***Continued*** |  |  |
| --- | --- | --- |
| **Plasmid, phage or Strain** | **Relevant genotype or feature** | **Source or reference** |
| **Phage** |  |  |
| Δ*lsr2*/*MSMEG_4732*Ci | Δ*lsr2* with pLJR962-*MSMEG_4732* | This study |
| Δ*lsr2*/*MSMEG_4733*Ci | Δ*lsr2* with pLJR962-*MSMEG_4733* | This study |
| Δ*lsr2*/*MSMEG_4735*Ci | Δ*lsr2* with pLJR962-*MSMEG_4735* | This study |
| Δ*lsr2*/*MSMEG_4737*Ci | Δ*lsr2* with pLJR962-*MSMEG_4737* | This study |
| A1SD1 |  | This study |
| K4JX5 |  | This study |
| K3GX1 |  | This study |
| G1HA1 |  | This study |
| L1HA1 |  | This study |
| L2JS15 |  | This study |
| I1GX4 |  | This study |
| F1GX13 |  | This study |
| C1GX3 |  | This study |

**References**

1. Zeng J, Zhang L, Li Y, Wang Y, Wang M, Duan X, et al. Over-producing soluble protein complex and validating protein-protein interaction through a new bacterial co-expression system. Protein Expr. Purif. 2010;69:47-53.
2. Li X, Long X, Chen L, Guo X, Lu L, Hu L, et al. Mycobacterial phage TM4 requires a eukaryotic-like Ser/Thr protein kinase to silence and escape anti-phage immunity. Cell Host Microbe 2023;31:1469-1480.
