# Supplementary material for: Insertion sequence transposition activates antimycobacteriophage immunity through an lsr2‐silenced lipid metabolism gene island
Source: mLife. 2024 Mar 26;3(1):87–100. doi: 10.1002/mlf2.12106 (PMC11139207; doi:10.1002/mlf2.12106)
Supplement: Supplementary file 6 — Supporting information. [file MLF2-3-87-s003.docx]

**Table S5. PCR primers used in this study.**

| **Primers** **name** | **Sequence 5'-3'** | | **Usage** | | |
| --- | --- | --- | --- | --- | --- |
| MSMEG_6092-F | ATATGCGGCCGCATGGCAAAGAAAGTGACCGT | | Cloned to pMV261 | | |
| MSMEG_6092-R | CGCGTCTAGACTAAGTTGCCGCGTGGAATG | | Cloned to pMV261 | | |
| MSMEG_6092-F | ATAT GGATCCATGGCAAAGAAAGTGACCGT | | Cloned and expression | | |
| MSMEG_6092-R | ATAT CTCGAGCTAAGTTGCCGCGTGGAATG | | Cloned and expression | | |
| MSMEG_5860-F | ATATGAATTCGCGTGTCCAGCGATGCAGTG | | Cloned and expression | | |
| MSMEG_5860-R | AGATTCTAGATC AT CGGTCCTCTCCCAGGA | | Cloned and expression | | |
| KoMSMEG_6092UP-F | ACTGCAGTCCATATGGATATCCTTAATTAAGGTTGCGGCCGAACTGGTCGAGCAC | | MSMEG_6092 knockout | | |
| KoMSMEG_6092UP-R | AGAAGGGTCGGTGCCCGGGACGGAAGGTTGCCAGCACGCCGGGCC | | MSMEG_6092 knockout | | |
| KoMSMEG_6092DN-F | TTGCGGCAGCGTGAAGCTAGCATATCAGGCTTACGGTGACTACAAC | | MSMEG_6092 knockout | | |
| KoMSMEG_6092DN-R | ATATCAGGCTTACGGTGACTACAACGATTC | | MSMEG_6092 knockout | | |
| KoS1-F | ACTGCAGTCCATATGGATATCCTTAATTAAGACATCCACGAGTCGATCCGCGAACT | | S1 knockout | | |
| KoS1-R | GCAACACAATTTTGAGTCAATCACCCTTGCGAAAGCGCCGCGACA | | S1 knockout | | |
| KoS1-F | CGGCGCTTTCGCAAGGGTGATTGACTCAAAATTGTGTTGCTCCTGT | | S1 knockout | | |
| KoS1-R | TTGCGGCAGCGTGAAGCTAGCCGCGACGTCGCTCTCACCGTCGTGCA | | S1 knockout | | |
| KoS2-F | ACTGCAGTCCATATGGATATCCTTAATTAAAACGACGCGGGGCCCAGGGCGCTCC | | S2 knockout | | |
| KoS2-R | TGGCGGTCTCCCGTGCGTGAATGCTGGCCTTCATCACCACCTTGC | | S2 knockout | | |
| KoS2-F | GTGGTGATGAAGGCCAGCATTCACGCACGGGAGACCGCCAGCCCG | | S2 knockout | | |
| KoS2-R | TTGCGGCAGCGTGAAGCTAGCTGGCCGGCCACGGCCTGAAACTGAC | | S2 knockout | | |
| KoS3-F | ACTGCAGTCCATATGGATATCCTTAATTAATCGGTACGCAACAACGACCGCTCAT | | S3 knockout | | |
| KoS3-R | GCGCCAAGCGCTCTCAACATGTGTGTGCGCAGTTGCCGGGCGCGG | | S3 knockout | | |
| KoS3-F | CCCGGCAACTGCGCACACACATGTTGAGAGCGCTTGGCGCCCTGC | | S3 knockout | | |
| KoS3-R | TTGCGGCAGCGTGAAGCTAGCCATCGCGTTCGTCGACGGAATCCAC | | S3 knockout | | |
| KoMSMEG_4727-4737UP-F | ACTGCAGTCCATATGGATATCCTTAATTAAGCCATTGCGGGCAAACATAATTGAC | | MSMEG_4727-4737 knockout | | |
| KoMSMEG_4727-4737UP -R | ATGACGCGATTTATGACGCGAGCTTCCCCTACTAATGACGCGATC | | MSMEG_4727-4737 knockout | | |
| KoMSMEG_4727-4737DN -F | CGTCATTAGTAGGGGAAGCTCGCGTCATAAATCGCGTCATCACCC | | MSMEG_4727-4737 knockout | | |
| KoMSMEG_4727-4737DN -R | TTGCGGCAGCGTGAAGCTAGCGCACACGCGTACGAGACCACACACT | | MSMEG_4727-4737 knockout | | |
| KoMSMEG_4728-4732UP-F | ACTGCAGTCCATATGGATATCCTTAATTAAATGAACTCGTCGAGCACGACTGGGC | | MSMEG_4728-4732 knockout | | |
| KoMSMEG_4728-4732UP-R | CGTGTCGACGATCTAGCGGAGGTTCTACTCCTCCCTTCGATGACT | | MSMEG_4728-4732 knockout | | |
| KoMSMEG_4728-4732DN-F | TCGAAGGGAGGAGTAGAACCTCCGCTAGATCGTCGACACGCCGCT | | MSMEG_4728-4732 knockout | | |
| KoMSMEG_4728-4732DN-R | TTGCGGCAGCGTGAAGCTAGCATGTGGAGCGCAGTCCTGGTACTCG | | MSMEG_4728-4732 knockout | | |
| KoMSMEG_4727-4732UP-F | ACTGCAGTCCATATGGATATCCTTAATTAAGCCATTGCGGGCAAACATAATTGAC | | MSMEG_4727-4732 knockout | | |
| KoMSMEG_4727-4732UP-R | CGTGTCGACGATCTAGCGGAAGCTTCCCCTACTAATGACGCGATC | | MSMEG_4727-4732 knockout | | |
| KoMSMEG_4727-4732DN-F | CGTCATTAGTAGGGGAAGCTTCCGCTAGATCGTCGACACGCCGCT | | MSMEG_4727-4732 knockout | | |
| KoMSMEG_4727-4732DN-R | TTGCGGCAGCGTGAAGCTAGCCCGCCAAGAAATAGCGTGGTGTGAT | | MSMEG_4727-4732 knockout | | |
| KoMSMEG_4733-4737UP-F | ACTGCAGTCCATATGGATATCCTTAATTAAGAACTACAACATGGGCCACAACCTC | | MSMEG_4733-4737 knockout | | |
| KoMSMEG_4733-4737UP-R | ATGACGCGATTTATGACGCGCGGATCAGCGGTCGCACAGCCAGCG | | MSMEG_4733-4737 knockout | | |
| KoMSMEG_4733-4737DN-F | GCTGTGCGACCGCTGATCCGCGCGTCATAAATCGCGTCATCACCC | | MSMEG_4733-4737 knockout | | |
| KoMSMEG_4733-4737DN-R | TTGCGGCAGCGTGAAGCTAGCGCACACGCGTACGAGACCACACACT | | MSMEG_4733-4737 knockout | | |
| KoMSMEG_4728UP-F | ACTGCAGTCCATATGGATATCCTTAATTAAATGAACTCGTCGAGCACGACTGGGC | | MSMEG_4728 knockout | | |
| KoMSMEG_4728UP-R | CTGGAATCGCCGAACCTCACGGTTCTACTCCTCCCTTCGATGACT | | MSMEG_4728 knockout | | |
| KoMSMEG_4728DN-F | TCGAAGGGAGGAGTAGAACCGTGAGGTTCGGCGATTCCAGCATGA | | MSMEG_4728 knockout | | |
| KoMSMEG_4728DN-R | TTGCGGCAGCGTGAAGCTAGCAACGCGGCGACCAGCGAGTGGCTGT | | MSMEG_4728 knockout | | |
| KoMSMEG_4731UP-F | ACTGCAGTCCATATGGATATCCTTAATTAACGTCAGACTGCTGCGCCGGCGCGGC | | MSMEG_4731 knockout | | |
| KoMSMEG_4731UP-R | GCGTTTTCGTTCCCATGGTGGGGGGTGTCACCTTTCCTATTCGAT | | MSMEG_4731 knockout | | |
| KoMSMEG_4731DN-F | ATAGGAAAGGTGACACCCCCCACCATGGGAACGAAAACGCCGGTC | | MSMEG_4731 knockout | | |
| KoMSMEG_4731DN-R | TTGCGGCAGCGTGAAGCTAGCTGCGGATCAATTGCCGCACCGCGTG | | MSMEG_4731 knockout | | |
| KoMSMEG_4733UP-F | ACTGCAGTCCATATGGATATCCTTAATTAAGAACTACAACATGGGCCACAACCTC | | MSMEG_4733 knockout | | |
| KoMSMEG_4733UP-R | AAGAAATAGCGTGGTGTGATCGGATCAGCGGTCGCACAGCCAGCG | | MSMEG_4733 knockout | | |
| KoMSMEG_4733DN-F | GCTGTGCGACCGCTGATCCGATCACACCACGCTATTTCTTGGCGG | | MSMEG_4733 knockout | | |
| KoMSMEG_4733DN-R | TTGCGGCAGCGTGAAGCTAGCTGGGACAACGCGATCGAGGATGTCA | | MSMEG_4733 knockout | | |
| KoMSMEG_4734UP-F | ACTGCAGTCCATATGGATATCCTTAATTAATGTGCGACCGCTGATCCGCTAGATC | | MSMEG_4734 knockout | | |
| KoMSMEG_4734UP-R | GGATTTCGATGCGCGTAGCGCGTGGTGTGATATGTGGAGCGCAGT | | MSMEG_4734 knockout | | |
| KoMSMEG_4734DN-F | GCTCCACATATCACACCACGCGCTACGCGCATCGAAATCCTTCCC | | MSMEG_4734 knockout | | |
| KoMSMEG_4734DN-R | TTGCGGCAGCGTGAAGCTAGCCCATCTCATCATCATCGTCGGAAAT | | MSMEG_4734 knockout | | |
| KoMSMEG_4737UP-F | ACTGCAGTCCATATGGATATCCTTAATTAAAATCCCCGCATGCAAACGGCTCACG | | MSMEG_4737 knockout | | |
| KoMSMEG_4737UP-R | ATGACGCGATTTATGACGCGCCACATCACGCCGACCGGTCGAGGG | | MSMEG_4737 knockout | | |
| KoMSMEG_4737DN-F | GACCGGTCGGCGTGATGTGGCGCGTCATAAATCGCGTCATCACCC | | MSMEG_4737 knockout | | |
| KoMSMEG_4737DN-R | TTGCGGCAGCGTGAAGCTAGCGCACACGCGTACGAGACCACACACT | | MSMEG_4737 knockout | | |
| MSMEG_4727Pro-F | CGCGGTACCAGATCTTTAAA AACTTTGAAAAGGTACTGTTACCGGGT | | Cloned to pMV261 | | |
| MSMEG_4727Pro-R | TCCATCGCACTGACGCGGACAAGCTTCCCCTACTAATGACGCGATC | | Cloned to pMV261 | | |
| MSMEG_4728-4729-F: | GCGTCATTAGTAGGGGAAGCT TGTCCGCGTCAGTGCGATGGACAT | | Cloned to pMV261 | | |
| MSMEG_4728-4729-R: | TGTCGCGGCCGAGTTCAGCATGCGAGC | | Cloned to pMV261 | | |
| MSMEG_4730-4732-F: | CCGAACGGGCACGTGCTCGCATGCTGA | | Cloned to pMV261 | | |
| MSMEG_4730-4732-R: | TTAACTACGTCGACATCGAT TCAGCGGTCGCACAGCCAGCGCATCACG | | Cloned to pMV261 | | |
| MSMEG_4728-F | CGCG GAATTCATGTTCGAGCTGACCGACAT | | Cloned to pMV261 | | |
| MSMEG_4728-R | CGCG TCTAGATCACGCTGATCTGAGATCGA | | Cloned to pMV261 | | |
| MSMEG_4731-F | CGCG GAATTCATGACCCGCAGTTCTGTCAT | | Cloned to pMV261 | | |
| MSMEG_4731-R | ATAT TCTAGACTACGCGTGGACGAGGCGGG | | Cloned to pMV261 | | |
| MSMEG_4733-F | AGCG GGATCCATGTGGAGCGCAGTCCTGGT | | Cloned to pMV261 | | |
| MSMEG_4733-R | CGCG AAGCTTCTAGATCGTCGACACGCCGC | | Cloned to pMV261 | | |
| MSMEG_4734-F | ATAG GGATCCATGCGCGACGTTCGCGTGGG | | Cloned to pMV261 | | |
| MSMEG_4734-R | CGCG AAGCTTCTATTTCTTGGCGGCCGACG | | Cloned to pMV261 | | |
| MSMEG_4737-F: | ATATGAATTCGTGTGTGCGCAGTTGCCGGG | | Cloned to pMV261 | | |
| MSMEG_4737-R: | ATCGTCTAGATCATGACGCGTGTGGTCCGA | | Cloned to pMV261 | | |
| MSMEG_4733-4737-F | CGCGGTACCAGATCTTTAAA TTGCTAAAACTCCTCGAGCATGTGA | | Cloned to pMV261 | | |
| MSMEG_4733-4737-R | TTAACTACGTCGACATCGAT CTAGATCGTCGACACGCCGCTGGCC | | Cloned to pMV261 | | |
| MSMEG_4727(S1) pro-F | AACTTTGAAAAGGTACTGTT | | Cloned | | |
| MSMEG_4727(S1) pro-R | GGAGGAGAGCCTCCCATAGC | | Cloned | | |
| MSMEG_4735(S2) pro-F | GGCGGCGGGAAGTCGACCGCGACGAACACG | | Cloned | | |
| MSMEG_4735(S2) pro-R | TCGGTGACGCGGAGCAAACCCACTGTGGTT | | Cloned | | |
| MSMEG_4737(S3) pro-F | GAAATAGCGTGTGCCCAAGAGGGTTCCGCC | | Cloned | | |
| MSMEG_4737(S3) pro-R | TTGCTAAAACTCCTCGAGCATGTGAAATTT | | Cloned | | |
| MSMEG_5860pro-F | CGAACACGCCTTTCCGGCCTGCGACGAGTTT | | Cloned | | |
| MSMEG_5860pro-R | AGCATCTCGATGCCCGCGGTGAGCACCTTGC | | Cloned | | |
| MSMEG_6092qRT-F | CGATGATTTCGACGGTGAG | | qRT-PCR | | |
| MSMEG_6092qRT-R | ACCCACTGCTTCAGATCGTT | | qRT-PCR | | |
| MSMEG_4727qRT-F | CCAAGGTCTACCGCATGGC | | qRT-PCR | | |
| MSMEG_4727qRT-R | CCAGGCTGGAGAACTCGAG | | qRT-PCR | | |
| MSMEG_4728qRT-F | GGACAGCCAGTGCGACATG | | qRT-PCR | | |
| MSMEG_4728qRT-R | ATCCCGAAGCGGAAGCAG | | qRT-PCR | | |
| MSMEG_4729qRT-F | AGAAGAACTGGGCGACTGG | | qRT-PCR | | |
| MSMEG_4729qRT-R | CGACGGTTTCACCCTTGA | | qRT-PCR | | |
| MSMEG_4730qRT-F | TCTGGGCGGGCTGTTCTT | | qRT-PCR | | |
| MSMEG_4730qRT-R | GTTCTGGGCATCGAGCTGATAG | | qRT-PCR | | |
| MSMEG_4731qRT-F | CGCTGTCGGTGCCATTC | | qRT-PCR | | |
| MSMEG_4731qRT-R | CTGCGGTTCGAGATATTCG | | qRT-PCR | | |
| MSMEG_4732qRT-F | CAACATGGGCCACAACCTC | | qRT-PCR | | |
| MSMEG_4732qRT-R | CGTAGCGTGCGAACACTTCT | | qRT-PCR | | |
| MSMEG_4733qRT-F | TCCCGCTGGTGGGTTATCTG | | qRT-PCR | | |
| MSMEG_4733qRT-R | CAGCATCAGGACACCGAACAAT | | qRT-PCR | | |
| MSMEG_4734qRT-F | GACGAGTGCGACATCAACGA | | qRT-PCR | | |
| MSMEG_4734qRT-R | GGCGGTCAAGATGAACGAGA | | qRT-PCR | | |
| MSMEG_4735qRT-F | CAAGGGCACCAAGAACG | | qRT-PCR | | |
| MSMEG_4735qRT-R | GCCGCAATAACGATAGAAGT | | qRT-PCR | | |
| MSMEG_4736qRT-F | CACGGCCTGAAACTGACA | | qRT-PCR | | |
| MSMEG_4736qRT-R | CGAGACCACCCGAAACC | | qRT-PCR | | |
| MSMEG_4737qRT-F | CTGAAGCTGACCGCAACG | | qRT-PCR | | |
| MSMEG_4737qRT-R | CGAGACCACCCGAAACC | | qRT-PCR | | |
| K4JX5qRT-F | GTCGCTCAATCCGCACTTCCA | | qRT-PCR | | |
| K4JX5qRT-R | GCCTCGGGCACGCTGTAGAAA | | qRT-PCR | | |
| sigA-F | CGAGGAAGAAGAAGCTGATG | | qRT-PCR | | |
| sigA-R | CGTCTTTGCGTGCCTGTC | | qRT-PCR | | |
| 16SrRNART-F | GATACGGGCAGACTAGAGTA | | qRT-PCR | | |
| 16SrRNART-R | GGGTATCTAATCCTGTTCGC | | qRT-PCR | | |
|  | |  | |  |  |
