# Supplementary material for: Insertion sequence transposition activates antimycobacteriophage immunity through an lsr2‐silenced lipid metabolism gene island
Source: mLife. 2024 Mar 26;3(1):87–100. doi: 10.1002/mlf2.12106 (PMC11139207; doi:10.1002/mlf2.12106)
Supplement: Supplementary file 7 — Supporting information. [file MLF2-3-87-s001.docx]

**Table S6. Sequence information of sgRNAs used for CRISPRi assays in this study.**

| **Primers name** | | **sgRNA sequence 5'-3'** |
| --- | --- | --- |
| MSMEG_0399Ci-F | | GGGACACCGAAAACCACCCGCCAC |
| MSMEG_0399Ci-R | | AAACGTGGCGGGTGGTTTTCGGTG |
| MSMEG_1238Ci-F | | GGGATAACTCGTCATCAGCACGAC |
| MSMEG_1238Ci-R | | AAACGTCGTGCTGATGACGAGTTA |
| MSMEG_1254Ci-F | | GGGATGCCCGCACCAATCGCACAC |
| MSMEG_1254Ci-R | | AAACGTGTGCGATTGGTGCGGGCA |
| MSMEG_2148Ci-F | | GGGAACCGGTAGCGGTATTTTCGG |
| MSMEG_2148Ci-R | | AAACCCGAAAATACCGCTACCGGT |
| MSMEG_2303Ci-F | | GGGAGCGCCGACGAGCCAGCCGCC |
| MSMEG_2303Ci-R | | AAACGGCGGCTGGCTCGTCGGCGC |
| MSMEG_2340Ci-F | | GGGAGGTGGTGCCGCCGCTGCGGC |
| MSMEG_2340Ci-R | | AAACGCCGCAGCGGCGGCACCACC |
| MSMEG_5029Ci-F | | GGGAATGTTGTGGTGTTCGGCGAC |
| MSMEG_5029Ci-R | | AAACGTCGCCGAACACCACAACAT |
| MSMEG_5393Ci-F | | GGGATCCTCCGGGGTGGCCTCGGC |
| MSMEG_539 Ci-R | | AAACGCCGAGGCCACCCCGGAGGA |
| MSMEG_5583Ci-F | | GGGACCCACCGCAGCACCTCATCC |
| MSMEG_5583Ci-R | | AAACGGATGAGGTGCTGCGGTGGG |
| MSMEG_6022Ci-F | | GGGACGGCAGCAATCGCGACCGTG |
| MSMEG_6022Ci-R | | AAACCACGGTCGCGATTGCTGCCG |
| MSMEG_6057Ci-F | | GGGAAGGATGTTCGGGGTCGTGTA |
| MSMEG_6057Ci-R | | AAACTACACGACCCCGAACATCCT |
| MSMEG_6090Ci-F | | GGGAGGGCGACGCTCTGGTGAGCC |
| MSMEG_6090Ci-R | | AAACGGCTCACCAGAGCGTCGCCC |
| MSMEG_6092Ci-F | | GGGAGCAACTTCGCGGCGTTTTTT |
| MSMEG_6092Ci-R | | AAACAAAAAACGCCGCGAAGTTGC |
| MSMEG_6150Ci-F | | GGGATCCGTCGAGGGGGAGATCAG |
| MSMEG_6150Ci-R | | AAACCTGATCTCCCCCTCGACGGA |
| MSMEG_4727Ci-F | | GGGAGCCGTCAGGGCCTCGGTGAC |
| MSMEG_4727Ci-R | | AAACGTCACCGAGGCCCTGACGGC |
| MSMEG_4728Ci-F | | GGGAGTGCGCTGGATGATCCCGAA |
| MSMEG_4728Ci-R | | AAACTTCGGGATCATCCAGCGCAC |
| MSMEG_4729Ci-F | | GGGATGGCCGGTGGCGCGCTGCCC |
| **Continued** | | |
| **Primers name** | | **sgRNA sequence 5'-3'** |
| MSMEG_4729Ci-R | | AAACGGGCAGCGCGCCACCGGCCA |
| MSMEG_4730Ci-F | | GGGAGGCAGGTCGTCGGGAGATCC |
| MSMEG_4730Ci-R | | AAACGGATCTCCCGACGACCTGCC |
| MSMEG_4731Ci-F | | GGGAGACGGCGCGATGTTGTCGGG |
| MSMEG_4731Ci-R | | AAACCCCGACAACATCGCGCCGTC |
| MSMEG_4732Ci-F | | GGGAGTAACCCTGCGCCAGCACCT |
| MSMEG_4732Ci-R | | AAACAGGTGCTGGCGCAGGGTTAC |
| MSMEG_4735Ci-F | | GGGAAACGACGTGCCGCAATAACG |
| MSMEG_4735Ci-R | | AAACCGTTATTGCGGCACGTCGTT |
| MSMEG_4737Ci-F | | GGGACCGAAAGCTCCCGCACCGCC |
| MSMEG_4737Ci-R | | AAACGGCGGTGCGGGAGCTTTCGG |
| MSMEG_2554Ci-F | | GGGATGGGATGCGACGGACCGTCG |
| MSMEG_2554Ci-R | | AAACCGACGGTCCGTCGCATCCCA |
| MSMEG_2982Ci-F | | GGGATGGTTGGTGGTGGCGCCGGT |
| MSMEG_2982Ci-R | | AAACACCGGCGCCACCACCAACCA |
| MSMEG_4533Ci-F | | GGGACGGGTGACGTCGGGTTCGAC |
| MSMEG_4533Ci-R | | AAACGTCGAACCCGACGTCACCCG |
| MSMEG_5519Ci-F | | GGGAAGGGTGAAGTCGTGGATGCG |
| MSMEG_5519Ci-R | | AAACCGCATCCACGACTTCACCCT |
| MSMEG_5863Ci-F | | GGGAAGTTCGCTGTCGCTGGAACG |
| MSMEG_5863Ci-R | | AAACCGTTCCAGCGACAGCGAACT |
| MSMEG_5961Ci-F | | GGGATATTCGGCGAGACACGGCAA |
| MSMEG_5961Ci-R | | AAACTTGCCGTGTCTCGCCGAATA |
|  |  |  |
